# Sex-biased gene and microRNA expression in the developing mouse brain is associated with neurodevelopmental functions and neurological phenotypes

**DOI:** 10.1186/s13293-023-00538-3

**Published:** 2023-09-07

**Authors:** Susanna Szakats, Alice McAtamney, Hugh Cross, Megan J. Wilson

**Affiliations:** Developmental Genomics Laboratory, Department of Anatomy, School of Biomedical Sciences, University of Otago, P.O. Box 56, Dunedin, 9054 New Zealand

**Keywords:** Sex-bias, microRNA, Neurodevelopment, Neurodevelopmental disorder, Compensation, Brain development

## Abstract

**Background:**

Sex differences pose a challenge and an opportunity in biomedical research. Understanding how sex chromosomes and hormones affect disease-causing mechanisms will shed light on the mechanisms underlying predominantly idiopathic sex-biased neurodevelopmental disorders such as ADHD, schizophrenia, and autism. Gene expression is a crucial conduit for the influence of sex on developmental processes; therefore, this study focused on sex differences in gene expression and the regulation of gene expression. The increasing interest in microRNAs (miRNAs), small, non-coding RNAs, for their contribution to normal and pathological neurodevelopment prompted us to test how miRNA expression differs between the sexes in the developing brain.

**Methods:**

High-throughput sequencing approaches were used to identify transcripts, including miRNAs, that showed significantly different expression between male and female brains on day 15.5 of development (E15.5).

**Results:**

Robust sex differences were identified for some genes and miRNAs, confirming the influence of biological sex on RNA. Many miRNAs that exhibit the greatest differences between males and females have established roles in neurodevelopment, implying that sex-biased expression may drive sex differences in developmental processes. In addition to highlighting sex differences for individual miRNAs, gene ontology analysis suggested several broad categories in which sex-biased RNAs might act to establish sex differences in the embryonic mouse brain. Finally, mining publicly available SNP data indicated that some sex-biased miRNAs reside near the genomic regions associated with neurodevelopmental disorders.

**Conclusions:**

Together, these findings reinforce the importance of cataloguing sex differences in molecular biology research and highlight genes, miRNAs, and pathways of interest that may be important for sexual differentiation in the mouse and possibly the human brain.

**Supplementary Information:**

The online version contains supplementary material available at 10.1186/s13293-023-00538-3.

## Plain language summary

In biomedical research, understanding the differences between males and females is essential for understanding diseases that affect one sex more than the other. This study focused on gene expression and regulation differences between male and female mouse brains during development. We found that many microRNAs, small molecules that play a role in development were expressed differently between male and female brains. These differences could be important in understanding why males and females develop differently, particularly regarding neurodevelopmental disorders like ADHD, schizophrenia, and autism. We also found that some microRNAs that differed between males and females were located near genes associated with these disorders. Overall, the study highlights the importance of understanding sex differences in molecular biology research and provides insights into potential genes and pathways of interest for further study.

**Supplementary Information:**

The online version contains supplementary material available at 10.1186/s13293-023-00538-3.

## Highlights

- Understanding how sex chromosomes and hormones affect disease-causing mechanisms will illuminate the mechanisms underlying predominantly idiopathic sex-biased neurodevelopmental disorders, such as ADHD, schizophrenia, and autism.
- We demonstrated robust sex differences for some genes and miRNAs expressed in the embryonic mouse brain.
- This is the first study to use high-throughput sequencing to capture traditional RNA-seq data in parallel with small RNA-seq data, which allowed us to gain new insights into how sex differences in miRNA and mRNA targets interact.
- Sex-biased genes, miRNAs, and pathways in which they act are known to be involved in neurodevelopmental processes, emphasizing the importance of RNA in establishing sex differences in the prenatal mouse brain.

**Supplementary Information:**

The online version contains supplementary material available at 10.1186/s13293-023-00538-3.

## Background

The prevalence, symptoms, and severity of several brain disorders differ between males and females [1]. This disparity implies that sex influences the phenotype of disease-causing mechanisms [2]. Evidence of sex differences also emphasizes the need to study both sexes in biomedical research; however, much research is male-centric [3]. The need to address the origin of sex differences is paramount for many neurodevelopmental disorders (NDDs), including ADHD, schizophrenia, and autism, which are primarily idiopathic and where previously assumed sex biases are becoming increasingly criticized [4–9]. Decades of findings collected from male subjects have failed to account for sex differences in the phenotypic presentation of these disorders and have resulted in the systematic underdiagnosis of females [3]. Thus, investigating the origins of sex differences in tissues and organs can aid in understanding sex-biased diseases.

Biological sex determinants, consisting of the sex chromosome complement and hormonal milieu, act through various genetic and epigenetic mechanisms to drive gene expression changes in the brain [10]. These mechanisms include Y-linked genes, genes that escape X-inactivation, and gonadal hormones [10]. Furthermore, coordinated gene expression patterns drive cellular processes that comprise neurodevelopment, such as proliferation, migration, differentiation, apoptosis, and synaptogenesis [11]. Therefore, gene expression and regulation are a critical nexus by which sex influences the developing brain.

Regulation of gene expression is multi-faceted and includes non-coding RNA species such as microRNAs (miRNAs) [12]. These small RNAs are encoded within the genome and are initially transcribed from a primary transcript processed through various precursor forms to form a mature miRNA, a short transcript ~ 22 nucleotides in length [13]. miRNAs negatively regulate translation through complementary base pairing with target messenger RNAs (mRNAs), and the RNA-induced silencing complex (RISC) associated with mature miRNAs degrades the target mRNA or inhibits translation to reduce the amount of protein produced [14]. Thus, miRNAs can fine-tune the expression of at least two-thirds of the mammalian genome [15]. In addition, there is abundant evidence that miRNAs are essential for neurodevelopment [16] and that changes in their expression lead to functional changes that contribute to NDD etiology [17].

Several review articles discussing sex differences in neurodevelopment have illustrated how sex differences arise through gonadal hormones, sex chromosomes, and epigenetic mechanisms. Furthermore, they emphasized the lack of data on the role of miRNAs in establishing sex differences in the developing brain [10, 18, 19]. Nevertheless, all argue that miRNAs, with their transient expression, rapid evolution, and ability to regulate many target genes, are prime candidates for generating subtle differences between sexes during development.

The influence of sex on miRNA expression has been increasingly studied in the adult brain [20–27], but few studies have addressed sex-differential miRNA expression during development. Ziats et al. [28] demonstrated sex differences in miRNA expression in the human brain from before birth to adulthood. However, these findings are limited by the caveats of using post-mortem human tissues, specifically low sample numbers and poor tissue preservation, which may be insufficient to capture rapidly degrading neural miRNAs [29]. More systematic experiments using rodent neurodevelopmental models can overcome these limitations. However, the studies by Morgan et al., Murphy et al., Morgan et al., and McCarthy et al. [18, 30–32] demonstrated sex-biased miRNAs in the developing brain, focusing on highly sexually differentiated brain regions, such as the hypothalamus, and tended to use microarray technology. No systematic investigation of known and novel miRNAs in the developing mammalian brain has been undertaken. Previous studies have mainly focused on individual brain regions, thereby neglecting the overall sex differences that exist throughout the entire tissue [33]. Furthermore, previous studies have not investigated the embryonic phase of neurodevelopment, which is a critical and sensitive window for neurodevelopment [34]. These gaps illustrate the need to study sex-differential miRNA expression in the embryonic mouse brain.

The main objective of this study was to describe the expression patterns of genes and miRNAs in mouse brain tissues aged E15.5 using high-throughput sequencing. Differential expression analysis was validated for a subset of transcripts by RT-qPCR. To derive further meaning from these results, we integrated RNA-seq and small RNA-seq data to investigate miRNA–mRNA networks in the developing brain. Finally, we explored the possible consequences of sex-differential gene and miRNA expression using pathway analysis and relevant disease associations. Overall, this study established sex differences at the RNA level, at a critical stage of mouse brain development.

## Materials and methods

### Animal husbandry and tissue collection

Breeding and dissection of C57BL/6 wild-type mice were performed with the approval of the University of Otago Animal Ethics Committee to generate embryos at specific developmental time points. Mice were housed under standard conditions with ad libitum access to food and water. A mating pair was housed together for up to four nights and checked each morning for copulation plugs. After identifying a copulation plug, the dam and stud were separated, and the developmental stage was considered embryonic day 0.5 (E0.5). Embryo staging was confirmed by assessing limb morphology during tissue collection. Dams were culled by cervical dislocation to collect embryos at E15.5. Embryo dissections were performed in cold, sterile 70% PBS to collect whole brain tissue for RNA isolation and gonad tissue to determine the sex of each embryo in the presence (male) or absence (female) of the testicular cords.

### RNA isolation and purification

Individual brains that had been sexed were kept in sterile PBS on ice for immediate RNA isolation or stored in RNALater (Ambion) at − 20 °C. RNA was extracted using the Purelink RNA miniprep kit (Ambion) following the manufacturer’s instructions. An optional DNase treatment step was included in this protocol to prevent genomic contamination of the RNA samples. Manual homogenization was performed with a sterile needle tip before passing through an 18-gauge syringe and centrifuging for 2 min at 12,000×*g*. In the final step, RNA was eluted in 50 μL mqH_2_O and stored at − 20 °C. RNA was purified using ethanol precipitation. The RNA pellet was resuspended in 15 μL milliq H_2_O (mqH_2_O). The concentration of each sample was measured using a NanoDrop spectrophotometer. The purity of each sample was analyzed using the 260/230 and 260/280 ratios. RNA samples with ratios of 1.8–2.2 were considered to be of sufficient quality for downstream application.

### RNA-sequencing

Three biological replicates of RNA from E15.5, each consisting of pooled RNA from 2–3 individuals, were prepared for each sex. RNA from 2–3 individuals was pooled to counteract any variation derived from litter-based effects and minor variations in the timing of embryo growth that occur naturally, even within a litter. RNA integrity of each replicate was confirmed to be RIN > 8.0 with a Bioanalyser (Agilent Technologies). One μg RNA from each replicate was used to generate a Illumina TruSeq Stranded Total RNA Library according to the manufacturer’s instructions. NZGL sequenced TruSeq libraries on an Illumina HiSeq platform (125-bp sequence reads, paired-end).

Initially, the sequence reads were analyzed using Galaxy (v. 18.05) [35]. Next, Trimmomatic (v 0.32) [36] was used to remove indexes and trim low-quality sequences, and then FastQC (v. 0.11.6) [37] confirmed a sequence quality of phred > 30 across all trimmed reads. Trimmed sequence reads were aligned to the mouse reference genome (version mm9) using TopHat (v. 2.1.1) [38]. Cufflinks (v 2.2.1) [39] was then used to assemble and quantify transcript abundance against the mm9 reference genome for each sample. Then all Cufflinks outputs were merged using Cuffmerge (v 2.2.1.0) [39] to create a master transcriptome, and featureCounts (v 1.5.1) [40] was then used to quantify transcripts based on the Cuffmerge master transcriptome for each sample alignment. The Generate Count Matrix (Galaxy v 1.0) tool then combined the transcript count outputs from featureCounts for each sample into a matrix of read counts that could be subsequently used for differential count analysis.

Differential expression analysis was performed in R Studio (v 1.0.136) with DESeq2 (v. 1.18.1) [41]. The matrix generated by featureCounts was imported, transcripts with low read counts (less than 10) across all samples were removed, and inbuilt normalization strategies of each package were used to scale raw read counts to account for library size differences. The RUVr function from RUVseq (v 1.12.0) [42] was also used to account for batch variation. Differential expression analysis between male and female samples was performed using the exact test function, DESeq2. Transcripts were considered significantly different between the sexes if they met the following criteria: DESeq2, adjusted *p*-value < 0.05.

### RT-qPCR

Sex differences in gene expression were confirmed by RT-qPCR, using additional biological replicates. First, the RNA (500 ng) was reverse-transcribed with qScript (Bio-Rad) following the manufacturer’s instructions to generate cDNA and diluted 1 in 2 in mqH_2_O. Negative controls were generated using RNA that was not reverse-transcribed. Next, 1 μL of diluted cDNA was added to 5 μL SYBR Green MasterMix (ThermoFisher), 1.25 μL primer, and 2.75 μL mqH_2_O in a 96-well plate. Each sample was loaded in triplicate. Oligonucleotide primers were designed for the selected differentially expressed genes using IDT PrimerQuest (https://sg.idtdna.com/primerquest/Home/Index), and their specificity and efficiency were tested (Additional file 1: Table S1). The 96-well plate was loaded into a Viia7 PCR Machine (ThermoFisher) for the RT-qPCR reaction, which was incubated for the following thermal profile: 30 s at 95 °C, followed by 40 cycles of 95 °C for 5 s followed by 60 °C for 19 s, followed by a machine-programmed dissociation curve. Expression was normalized to the geometric mean of the reference genes, *ActB* and *Rpl37*, using 2^−ΔCt^, where ΔCt = Ct^gene^ − Ct^reference gene^.

### Small RNA-sequencing

Sample preparation was performed according to the RNA-sequencing protocol (above). One μg of RNA was used to create Illumina Truseq Small RNA Libraries, which underwent Illumina HiSeq sequencing (50 bp reads, single-end) to generate 20 M reads per sample. Sequencing data were processed using the miRDeep2 workflow [43]. First, cutadapt (v. 1.15) [44] was used to remove adaptor sequences (TGGAATTCTCGGGTGCCAAGG) from the reads, allowing for up to two sequence mismatches. The sequences were then quality filtered using FastQC software. Next, reads were mapped using Bowtie (v 1.2.1) [45] to predefined miRNA precursors and mature miRNA reference sequences (miRbase v.21 and mm9 reference genome, respectively) to determine the expression of known miRNAs. The quantification output for each sample was then combined into a matrix of all read counts for the known miRNAs. Finally, differential expression analysis was performed using RStudio with the same parameters as the RNA-sequencing methods.

### microRNA RT-qPCR

The MystiCq microRNA cDNA Synthesis Kit (Sigma) was used to validate sex differences detected by small RNA-sequencing. PolyA Tailing and cDNA synthesis reactions were carried out as per the manufacturer’s instructions using 1 μg RNA as input, with the addition of 0.5 μL of 5 nM *cel-miR-39* spike-in oligo to the PolyA Tailing reaction mix as an exogenous reference gene. Negative controls were generated using polyA-tailed RNA that did not contain reverse transcriptase added during cDNA synthesis. Next, 1 μL of microRNA cDNA (diluted 1 in 2 in mqH_2_O) was added to 5 μL SYBR Green MasterMix (ThermoFisher), 0.75 μL each primer, and 2.5 μL mqH_2_O in a 96-well plate, where the two primers consist of (a) a miRNA-specific forward primer designed in IDT PrimerQuest, and (b) a universal reverse primer provided in the MystiCq Kit. Primers were tested for specificity and efficiency (sequences provided in Additional file 1: Table S2). Each sample was loaded in triplicate. The 96-well plate was loaded into a Viia7 PCR Machine (ThermoFisher) for the RT-qPCR reaction with the following thermal profile: 2 min at 50 °C, 2 min at 95 °C, 40 cycles of 15 s each at 95 °C, 60 °C and 72 °C, followed by a machine-programmed dissociation curve. Expression was normalized to the reference gene *cel-miR-39* using the Pfaffl method to account for deviated primer efficiency^47^, where female samples were used as the calibrator.

### Integrated miRNA–mRNA analysis

Target genes of miRNAs that showed significant sex differences in small RNA-seq (DESeq2 adjusted *p*-value < 0.05) were identified using miRTarBase (v. 9.0) [46], with the requirement of experimental validation of the miRNA–target interaction. The transcript IDs of target genes were overlapped with transcripts that showed significant differences between males and females (RNA-seq, DESeq2 *p* adj. < 0.05) to determine which sex-biased miRNAs targeted sex-biased mRNAs.

### GO analysis

Four gene lists were submitted to ShinyGO (v 0.75) [47] to identify any enriched KEGG pathways and gene ontology terms with FDR < 0.05: (1) male-biased genes, (2) female-biased genes, (3) miRTarBase that identified target genes of male-biased miRNAs, and (4) miRTarBase that identified target genes of female-biased miRNAs, where miRTarBase is a miRNA target identification database (https://mirtarbase.cuhk.edu.cn/). Enrichment analysis was also performed directly on the miRNA lists using miEAA (v. 2.0) [48] to identify and conduct gene set enrichment analyses for male-biased and female-biased miRNAs in the mammalian ncRNA-disease repository (MNDR) database [49]. Over-representation analysis was used to identify significantly enriched categories, where ‘background’ was set to all miRNAs expressed in the E15.5 mouse brain (according to the small RNA-seq data).

### SNPs associated with miRNAs of interest

The association of miRNAs of interest with neurological phenotypes and disease traits was performed to predict the clinical relevance of seven miRNAs with sex-biased expression in the E15.5 mouse brain. Before single nucleotide polymorphism (SNP) identification, conservation of the miRNAs of interest was tested by aligning the mature sequence of the mouse miRNA (obtained from miRBase v. 22.1) against the human genome (hg38) using the BLAT [50]. Mining of SNP databases provided evidence of an association with the relevant phenotypes. Information from two SNP databases was added to the hg38 assembly in the UCSC genome browser: (1) SNPedia pages with manually typed text [51] and (2) the NHGRI-EBI Catalog of Published Genome-Wide Association Studies [52]. SNPs were identified ± 100 kb from the transcription start site of each miRNA of interest, which is considered a feasible range for *cis*-regulatory interactions [53]. All SNPs identified at the miRNA loci were manually curated to retain only those associated with relevant phenotypes or associations with neurological diseases. Database mining for SNPs was supplemented by a literature search.

### Statistical analysis

All data are presented as mean ± standard error of the mean (SEM). Sex differences measured by RT-qPCR were reported as log_2_ fold change relative to females, and significance was calculated using a one-sample *t*-test (*μ* = 0). Sequencing and RT-qPCR validation were compared using a linear correlation analysis. All statistical analyses were performed in the Prism 8.0 software (GraphPad Software) with *p* < 0.05 as the threshold for statistical significance.

### Data availability

Raw sequencing files for RNA-seq and small RNA-seq were deposited on NCBI GEO: GSE211816 (publically available as of February 2nd, 2023).

## Results

### Sex-biased gene expression in the E15.5 mouse brain

**Fig. 1:**
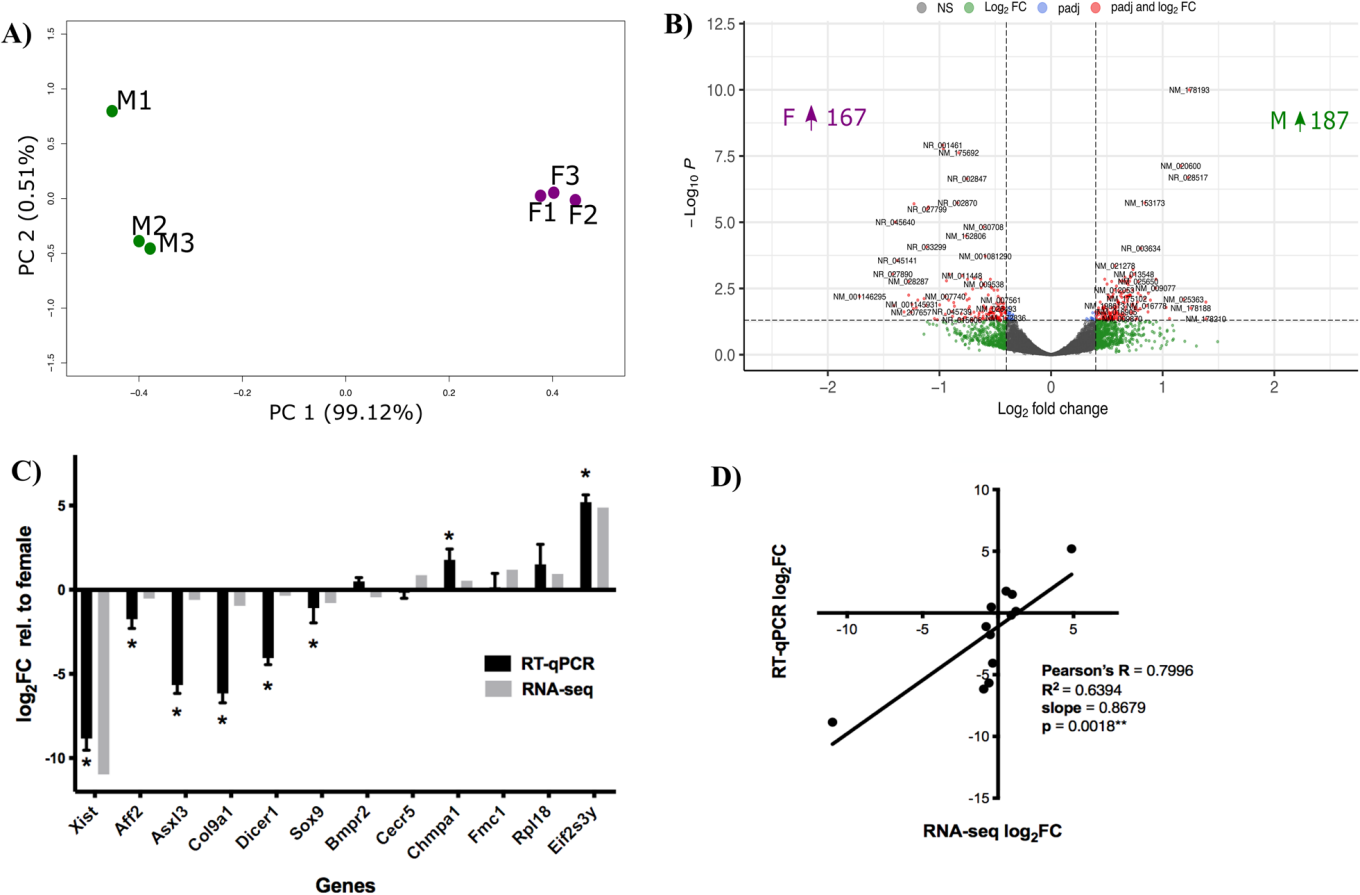
RNA-seq to identify sex-biased transcript expression. **A** PCA plot. The principal component analysis demonstrated clustering of samples and their relationships. Numbering indicates the biological replicate. **B** Volcano plot. The DESeq2 results were depicted by plotting the magnitude of the difference (*x*-axis: log_2_ fold change) against statistical significance (*y*-axis: − log_10_*P*). Each dot, representing one transcript, is color-coded as described in the key to indicate statistical significance (blue), magnitude of fold change (green), both (red), or neither (grey). Dotted lines indicate the thresholds for significance. **C** RT-qPCR. Graph of log_2_ fold change relative to females obtained from DESeq2 analysis of RNA-seq data (grey, *p adj. < 0.05) and calculated from 3 to 6 biological replicates of RT-qPCR (black, *p-value < 0.05, one-sample *t*-test, *µ* = 0). **D** Correlation analysis. Linear correlation between log_2_FC values from RNA-seq (*x*-axis) compared to RT-qPCR (*y*-axis) determined using Pearson’s *R*. *M* male, *F* females

Sex differences in gene expression were measured by performing differential expression analysis of RNA-seq data generated from *n* = 3/sex. Each replicate consisted of pooled RNA from 2 to 3 individuals at E15.5. This developmental stage was selected because it is a dynamic period for neurodevelopment and occurs after gonadal sex determination at ~ E12.0, meaning that circulating hormones such as testosterone (T) will be able to exert an effect on the brain. Sequencing reads of high quality across the sequence length (phred > 30) were mapped to the mm9 reference genome at a mean rate of ~ 80%, and yielded library sizes of > 30 M reads per sample (Additional file 1: Fig. S1). After normalization of RNA-seq libraries, PCA analysis demonstrated that samples clustered into males or females, as all variance between samples can be explained by sex (Fig. 1A). Differential expression analysis with DESeq2 identified 354 genes (*p* adj. < 0.05), with 187 upregulated in the male brain and 167 upregulated in the female brain at E15.5. The direction, magnitude, and significance of each of the 14,006 transcripts expressed in the E15.5 mouse brain are depicted in Fig. 1B. Complete RNA-seq differential expression analysis is available in Additional file 2: Data S1.

Differentially expressed transcripts were selected to validate the RNA-seq findings using RT-qPCR. The selected genes were a mixture of male-biased, female-biased, sex-linked, autosomal, or described in the literature as relevant to neurodevelopment. Sex-biased gene expression was replicated for most transcripts measured by RT-qPCR, with eight genes reaching statistical significance (Fig. 1C; *p* < 0.05*, one-sample *t*-test, *μ* = 0). A correlation analysis was also performed to determine consistency between RT-qPCR and RNA-seq measures by comparing the log_2_FC calculated by RNA-seq (DESeq2) against RT-qPCR. The linear regression model showed high concordance between the two methods (Fig. 1D; Pearson’s *R* = 0.7996, *p* = 0.0018**). Although the strength of the correlation is notably driven by very strong sex biases in *Xist* and *Eif2s3y*, a significant correlation remains after the exclusion of these data points (Pearson’s *R* = 0.70, *p* = 0.023*, data not shown). A comparable fold change between the two methods suggests that RNA-seq data reflect genuine sex differences in gene expression.

### Sex-biased miRNA expression in the E15.5 mouse brain

Sex differences in miRNA expression were measured by performing differential expression analysis of small RNA-seq data generated from *n* = 3/sex. Sequencing reads were of high quality across the sequence length (phred > 30), where the read length distribution peaked at 22 bp, the expected size of a mature miRNA (Additional file 1: Fig. S2B, C). A very high mapping rate of > 99% resulted in an average of 37M reads per library (Additional file 1: Fig. S2A). The miRNAs that had the highest read counts among all small RNA libraries included those with well-characterized neurodevelopmental functions (miR-9-5p, miR-125b-5p, miR-92a-3p, miR-181a-5p, miR-99a-5p, and let-7c-5p) (Additional file 1: Table S3).

**Fig. 2:**
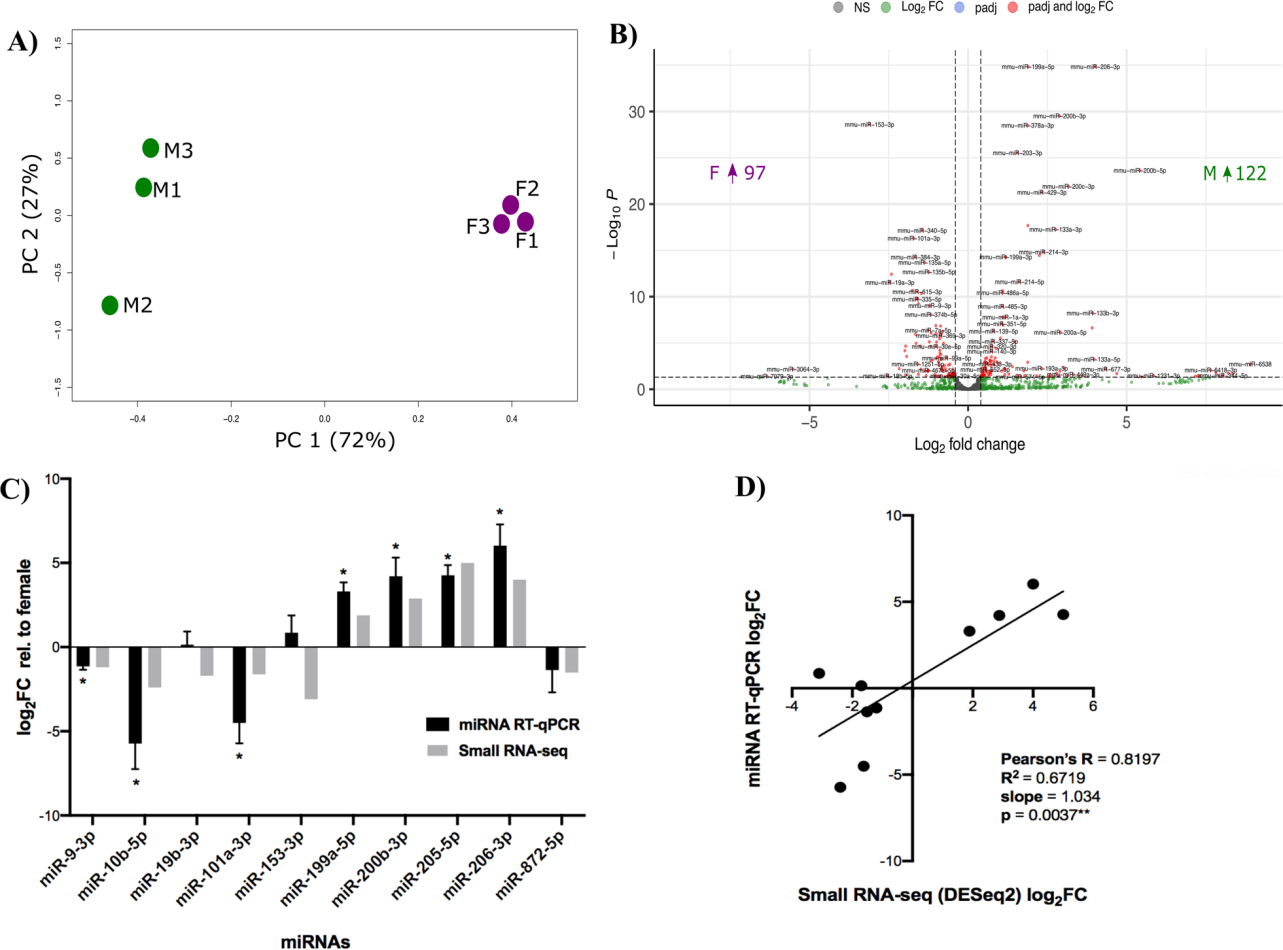
Small RNA-seq to identify sex-biased miRNA expression. **A** PCA plot. PCA plot shows the clustering between biological replicates and that most variation is between sexes. **B** Volcano plot. The DESeq2 results were depicted by plotting the magnitude of the difference (*x*-axis: log_2_ fold change) against statistical significance (*y*-axis: − log_10_*P*). Each dot representing one transcript is color-coded as described in the key. **C** miRNA RT-qPCR. Graph of log_2_ fold change relative to females obtained from DESeq2 analysis of RNA-seq data (grey, *p adj. < 0.05) and calculated from 3 to 6 biological replicates of RT-qPCR (black, *p-value < 0.05, one-sample *t*-test, *µ* = 0). **D** Correlation of miRNA RT-qPCR with DESeq2 analysis. Linear correlation between log_2_FC values from RNA-seq (*x*-axis) compared to RT-qPCR (*y*-axis) determined using Pearson’s *R*

After normalization of the small RNA-seq libraries, PCA analysis demonstrated that samples clustered by sex (Fig. 2A). Differential expression analysis identified 219 miRNAs (adjusted *p*-values. < 0.05), 122 male-biased and 97 female-biased (Fig. 2B). A complete list of the differentially expressed miRNAs is available in Additional file 3: Data S2. RT-qPCR validation was performed to replicate sex-biased miRNA expression in the E15.5 mouse brain. MiRNAs were selected for validation based on their described role in neurodevelopment according to the literature, also ensuring the selection of miRNAs with fold changes ranging from those with the greatest difference to those with a more modest magnitude. Significant differences were detected between males and females for seven of the ten miRNAs quantified by RT-qPCR (Fig. 2C): *miR-9-3p*, *miR-10b-5p*, *miR-101a-3p*, *miR-199a-5p*, *miR-200c-3p*, *miR-205-5p,* and *miR-206-3p* (*p* < 0.05*, one-sample *t*-test, Wilcoxon test correction, *n* = 5–7). Linear correlation analysis between the small RNA-seq results and the RT-qPCR showed a high concordance between the two methods (Fig. 2D; Pearson’s *R* = 0.82, *p* = 0.0037**).

### Integrating sex-biased mRNA and miRNA expression

**Fig. 3:**
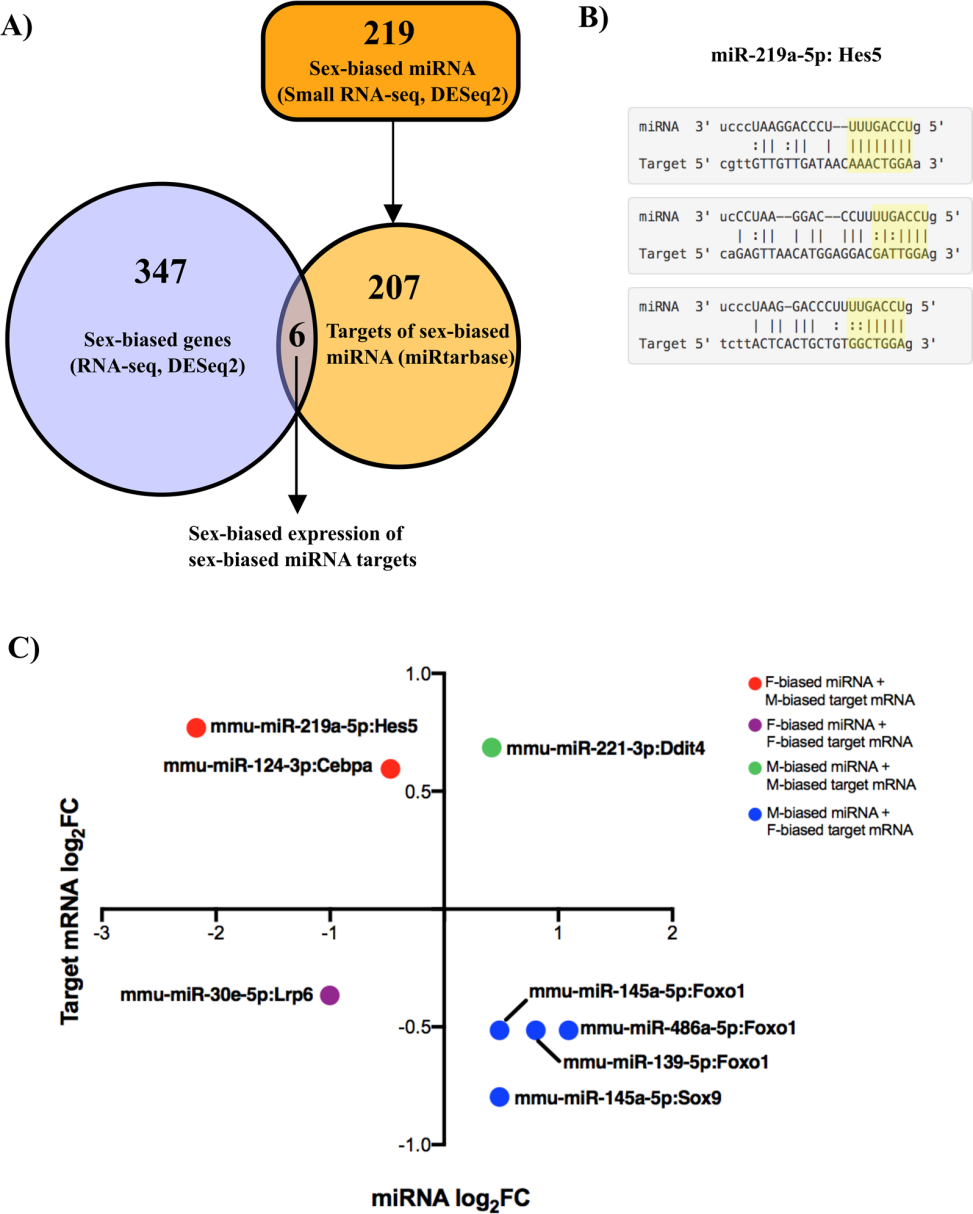
Sex-biased genes targeted by sex-biased miRNAs. **A** miRNA target identification process and Venn diagram showing transcripts common to sex-differential gene expression analysis and validated target genes of sex-biased miRNAs. **B** Example miRNA:target sequence pairing of *miR219a-5p:Hes5* complementary binding from miRanda [54] reported on miRtarbase. **C** Plot of 8 miRNA:target pairs using log_2_FC values from DESeq2 analysis of small RNA-seq and RNA-seq data

Having characterized sex-differential gene and miRNA expression in the developing mouse brain, we next wanted to integrate this information from these two datasets, as these two expression patterns do not exist independently but in concert. From 219 sex-biased miRNAs in the E15.5 mouse brain, miRtarbase identified 207 experimentally validated target genes. Only six transcripts were common among the 207 target genes and the sex-biased transcripts (Fig. 3A; RNA-seq, DESeq2 *p* adj. < 0.05). Given that miRNAs could have multiple target mRNAs, this resulted in eight possible interactions. For each possible pairing, it was confirmed that the mRNA 3′-UTR contains highly complementary sequences to their respective miRNA seed sequences (Fig. 3B). While stringent target prediction criteria yielded only a small dataset, most (6/8) of the miRNA:target pairs showed a negative correlation with their RNA-seq log_2_FC compared to their small RNA-seq log_2_FC (Fig. 3C), which is consistent with the degradation of mRNA targets by miRNA-mediated mechanisms.

### Predicted functions of sex-biased transcripts

Pathway analysis was conducted to determine which processes and pathways sex-biased genes and miRNAs act during brain development, suggesting the functional consequences of these sex differences on neurodevelopment. In addition, we performed enrichment analysis of sex-biased genes and target genes of sex-biased miRNAs to identify and compare the major biological functions between sexes.

**Fig. 4:**
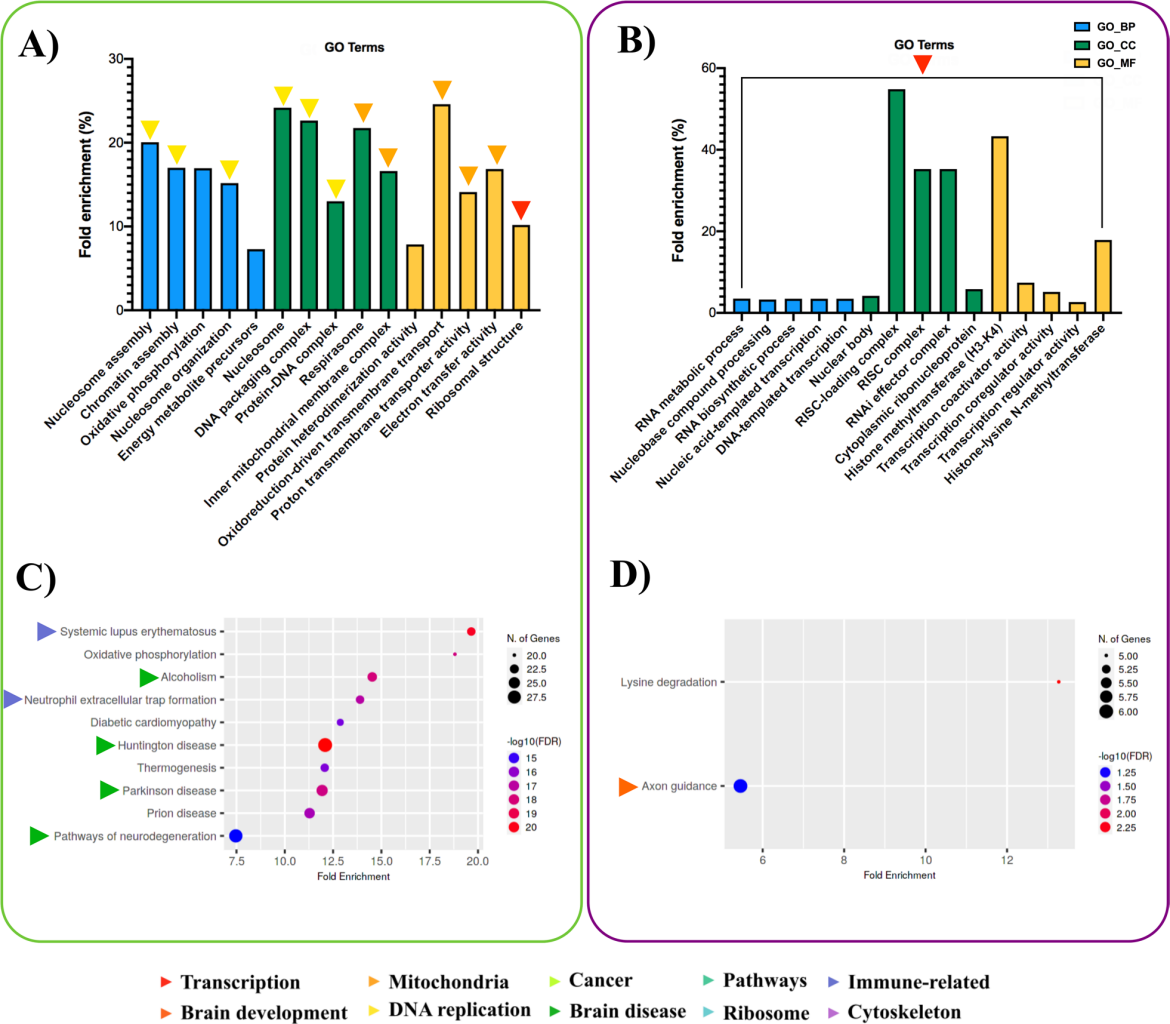
Top five enriched GO terms. Male-biased (**A**) and female-biased (**B**) genes in three categories: GO_BP (blue) = Gene Ontology_Biological Processes, GO_CC (green) = Gene Ontology_Cellular Compartment, GO_MF (yellow) = Gene Ontology_Molecular Function. Top 10 enriched KEGG pathways among **C** male and **D** female-biased genes. Dot plot color and size indicate − log_10_(FDR) for each pathway or GO term and the number of genes in each pathway, respectively. Colored arrows for each pathway name/GO term indicate manually curated functional clusters

Enrichment analysis of sex-biased genes (RNA-seq) and sex-biased miRNA target genes (small RNA-seq followed by miRTarBase) was performed against four commonly used gene sets [Gene Ontology (GO) Biological Processes, Cellular Compartment, Molecular Function, and KEGG pathways] via ShinyGO. The top five enriched terms ranked by fold enrichment are plotted in Fig. 4A, B, where colored arrows were used to annotate manually curated functional clusters. The 187 male-biased genes showed considerable enrichment for functions associated with DNA replication and mitochondrial function as well as multiple incidences of enrichment among pathways in brain disease, immune-related terms, and ribosomal function (Fig. 4C). In contrast, 167 female-biased genes were primarily associated with transcription and its regulation, with a notable enrichment of terms related to miRNA function under GO_CC (Fig. 4B). Only two KEGG pathways were enriched among female-biased genes, with axon guidance being highly relevant for brain development (Fig. 4D). Raw GO analysis for differentially expressed genes is available in Additional file 2: Data S1.

**Fig. 5:**
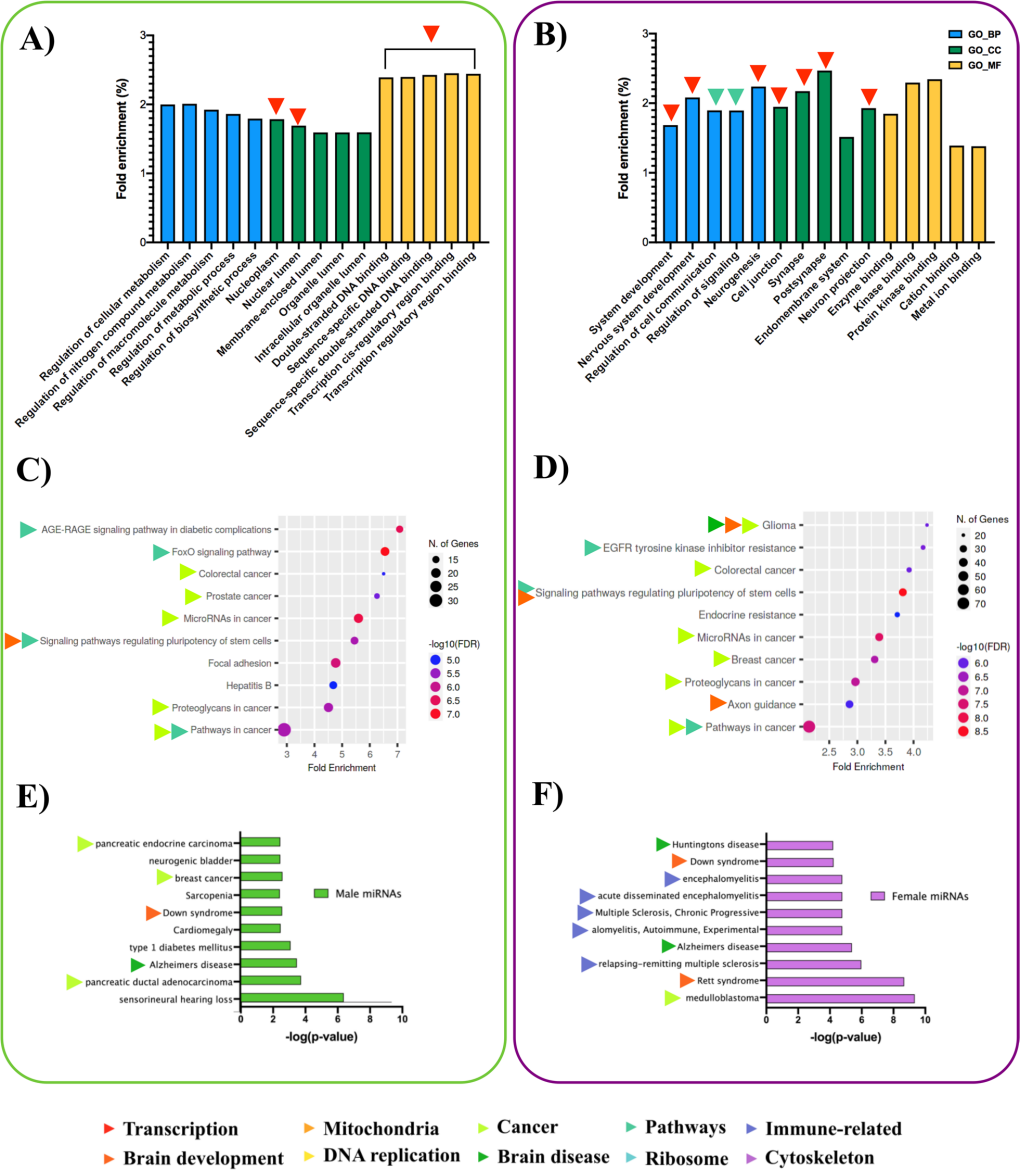
Top 5 enriched GO terms. Terms are shown for the target genes of male-biased (**A**) and female-biased (**B**) miRNAs in three categories: GO_BP (blue) = Gene Ontology_Biological Processes, GO_CC (green) = Gene Ontology_Cellular Compartment, GO_MF (yellow) = Gene Ontology_Molecular Function. Top 10 enriched KEGG pathways among the target genes of **C** male-biased and **D** female-biased miRNAs. Dot plot color and size indicate − log_10_(FDR) for each pathway or GO term, and the number of genes in each pathway, respectively. miEAA analysis of the top 10 disease terms for **E** male-biased and **F** female-biased miRNAs in MNDR database. Colored arrows by each pathway name/GO term indicate manually curated functional clusters

The enrichment analysis of sex-biased miRNAs was performed using target genes. Of the 122 male-biased miRNAs (small RNA-seq), miRTarBase identified 469 genes targeted by at least one miRNA. The most enriched terms among these 469 targets were predominantly related to transcription and brain development (Fig. 5A). The KEGG pathway findings included several signaling pathways and cancer-related categories (Fig. 5C). The 97 female-biased miRNAs yielded 1451 target genes according to miRTarBase. The most enriched function among the target genes was brain development (Fig. 5B). Many cancer-related KEGG pathways were also observed, but in contrast to the target genes of male-biased miRNAs, fewer enriched GO terms were related to transcription (Fig. 5D). In addition to functional enrichment analysis of miRNA target genes, we determined whether sex-biased miRNAs were enriched in the mammalian ncRNA-disease repository (MNDR) database. Among the significant MNDR hits, male-biased miRNAs showed brain diseases, such as Alzheimer’s disease, as well as several cancer-related terms that replicate the findings for male-biased miRNA targets (Fig. 5E). Female-biased miRNAs also showed significant enrichment for many neurological and NDDs (Rett syndrome, Alzheimer’s disease, Huntington’s disease) as well as immune-related diseases (Fig. 5F). Complete GO analysis for differentially expressed miRNAs is available in Additional file 3: Data S2.

Overall, functional prediction revealed 10 functional clusters that differed between sexes due to sex-biased gene and miRNA expression in the embryonic mouse brain: transcription, brain development, mitochondria, DNA replication, cancer, brain disease, signaling pathways, ribosome, immune-related, and cytoskeleton. The finding that transcriptional regulation and neurodevelopmental functions are the most prevalent enriched terms among sex-biased genes and miRNAs reinforces the importance of the RNA biology underlying sex differences in the developing brain.

### Sex-biased miRNAs associated with neurodevelopmental disorders

In addition to exploring the global consequences of sex-biased miRNA expression in the embryonic mouse brain via pathway analysis, we investigated the specific functions and possible human disease relevance of seven miRNAs of interest that showed 100% conservation of their mature sequences between mice and humans (Additional file 1: Fig. S4): *miR-9-3p*, *miR-10b-5p*, *miR-101a-3p*, *miR-199b-5p*, *miR-200b-5p*, *miR-205-5p*, and *miR-206-3p.* These seven miRNAs were considered sex-biased as they met the significance criteria used in the small RNA-seq analysis (*p* adj. < 0.05; Fig. 2B)*,* and sex-biased expression was replicated with RT-qPCR (*p* < 0.05*, one-sample *t*-test, Wilcoxon test correction; Fig. 2C). Here, we identified SNPs associated with neurological traits and disease phenotypes ± 100 kbp from each miRNA of interest and its paralogs using two publicly available SNP databases supplemented with relevant associations reported in the literature. A list of all identified SNPs can be found in Additional file 4: Data S3.

**Fig. 6:**
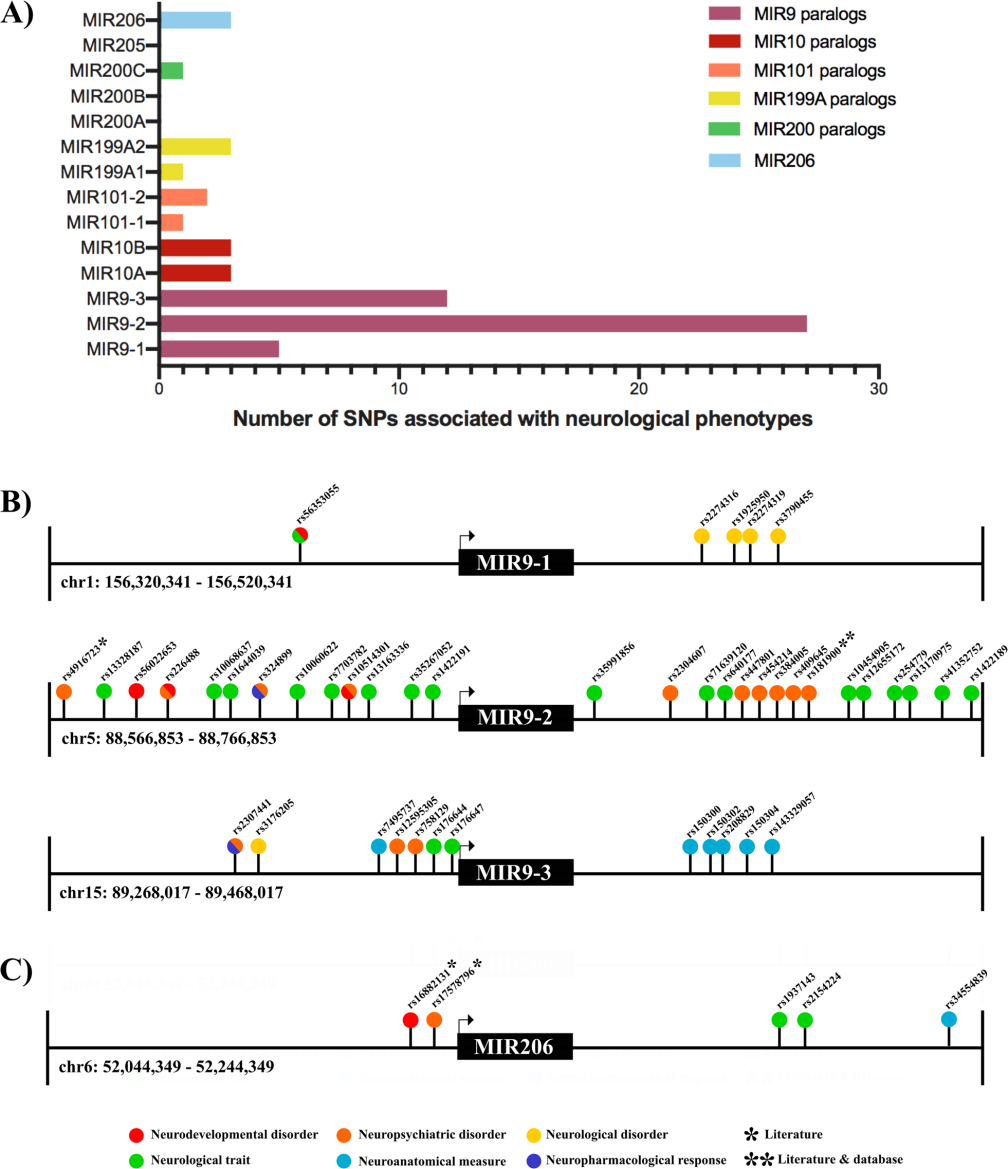
SNPs associated with neurological phenotypes ± 100 kbp sex-biased miRNAs. **A** The number of SNPs associated with a neurological phenotype is reported for each miRNA and key paralogs. Schematic representation of the regions surrounding human MIR9 paralogs 1–3 (**B**) and MIR206 (**C**). The SNPs were marked with their IDs in the approximate position relative to the miRNA gene. Color coding indicates manually curated functional groupings of SNP phenotypes. A single asterisk denotes an SNP identified in the literature, two asterisks denote SNPs identified in the literature and from mining databases, and no asterisks denotes an SNP identified in databases only

Mining publically available GWAS data revealed that some miRNAs tested showed no association with neurological phenotypes; most showed < 5, but MIR9 paralogs, particularly MIR9-2 and MIR9-3, were associated with a considerable number of relevant neurological phenotypes (Fig. 6A). MIR9-2 was associated with 27 SNPs representing 15 different phenotypes, and MIR9-3 with 12 SNPs and 6 phenotypes, with all traits reflecting neurological measures or neuropsychiatric disorders (Fig. 6B). Strikingly, the traits associated with MIR9-2 and MIR9-3 are predominantly neuropsychiatric disorders and those associated with mental illness. The literature reports that this cluster of disorders tends to be more prevalent in females [55], whereas our data showed female-biased *miR-9-3p* expression in the developing brain. Evidence for the sex-biased expression of an miRNA functioning in neurodevelopment [56], coinciding with sex-biased disease outcomes genetically associated with that miRNA locus, generates a promising new hypothesis for the mechanistic basis of female-biased neuropsychiatric disorders.

In contrast to the plethora of SNPs found in MIR9 loci using a database mining approach, other miRNAs of interest tended to be associated with fewer (0–5) neurological disease-related SNPs (Fig. 6A). To augment our database mining investigation, we searched the literature for additional evidence that sex-biased miRNAs may be associated with NDDs. This search revealed evidence for the association of *miR-9-3p* and *miR-206* with relevant neurological phenotypes. *MIR9* genes and nearby regions have been linked to multiple disorders including schizophrenia, ADHD, ASD, and MDD (Fig. 6B) [46, 57]. Additionally, MIR206 and the *miR-133/206* cluster had several significant associations with similar neurodevelopmental and/or neuropsychiatric disorders (schizophrenia, bipolar disorder, and ASD) (Fig. 6C) [58, 59]. These findings suggest the possible clinical relevance of sex-biased miRNAs in NDDs.

## Discussion

### Sex-biased expression is extensive among miRNAs

Sex differences in gene expression have previously been described in the mammalian brain at all stages of development, starting with neural stem cells (NSCs) [60], in the brain before sex determination [61], in the prenatal period, reaching maximum levels during puberty, and persisting in the adult brain [62, 63]. Our findings demonstrated significant differences in the expression levels of 272 transcripts, reinforcing sex differences in the mouse brain at E15.5. There is some consistency in the identity of sex-biased genes between the present study and previous publications, particularly for sex chromosome genes that are either Y-linked or have an X–Y homolog pair. We also demonstrated sex-biased expression of many autosomal genes and sex differences in the expression of specific transcript variants. However, many genes identified in our analysis have not been previously reported to be sex-biased in the brain. This difference is likely attributed to (1) dynamic temporal expression patterns, where sex differences vary considerably over time; thus, our findings differ from those of previously published studies conducted at different time points, and (2) regional variation observed between functionally diverse brain areas, where different studies have used different brain areas to study sex differences in gene expression. Overall, we report a sex bias of 272 of 14,006 transcripts expressed in the E15.5 mouse brain (~ 2%). This proportion is lower than some published findings, one of which reported up to 13% of the mouse brain transcriptome to be sex-biased [64]. The discrepancy between our data and previous studies could be due to the different cut-off criteria used and the use of different platforms (for example, RNA-seq vs. microarray), time point used (e.g., adult vs. embryo), or whole brain tissue that masks region-specific sex differences.

In contrast, the proportion of sex-biased miRNAs in the E15.5 mouse brain was considerably higher (219/932, or ~ 23%). Similar to sex-biased gene expression, our data replicated the broad concept of sex differences in somatic tissue. However, differences in the developmental stage, brain region, and species used in other studies make it difficult to make a direct comparison of the individual sex-biased miRNAs found here to the existing literature. The sex-biased expression of the following seven miRNAs was successfully validated by RT-qPCR: *miR-9-3p*, *miR-10b-5p*, *miR-101a-3p*, *miR-199a-5p*, *miR-200c-3p*, *miR-205-5p*, and *miR-206-3p*. Previous, studies have indicated that each of these miRNAs is a strong candidate to contribute to sex differences in the developing mouse brain, with numerous references to known roles in neurodevelopment [65–67], identification in studies investigating the mechanisms underlying NDDs [59, 68, 69], and a few examples of support for sex-biased expression in the brain [18, 29, 32, 70].

### Functions of sex-biased mRNA and miRNA in mouse neurodevelopment

Key findings from the functional enrichment analysis of sex-biased transcripts and miRNA expression provided insight into the possible functions of sex-biased genes and miRNAs in the E15.5 mouse brain. Although enrichment analysis was conducted in a way that generated redundant terms, the observation that ‘Transcription’ and ‘Brain development’ were the most frequently identified reassures us that the findings are relevant to the tissue of interest. Regulation of transcription is among the functions enriched by sex-biased genes and miRNAs, emphasizing the importance of RNA biology and post-transcriptional regulation of gene expression in the developing brain and reinforcing the premise of this investigation. Terms associated with brain development were also frequent among sex-biased miRNAs, providing further evidence that sex differences in miRNA expression may have functional consequences leading to sex differences in the brain.

Other vital functions identified by enrichment analysis include various aspects of cell growth and development (DNA replication, cancer, and signaling pathways), which could contribute to sex differences in brain size or the rate of brain development [71, 72]. However, the emphasis on cancer-related pathways likely reflects the extent of cell division occurring in the rapidly expanding embryonic brain. Furthermore, the enrichment of immune-related functions and mitochondria was intriguing. The former has been repeatedly shown to differ between the sexes [73], and the latter, with uniquely maternal inheritance patterns, is subject to unique evolutionary forces between the sexes [74]. The final functional category identified was related to brain disease. This group includes both neurodevelopmental and neurodegenerative disorders that are known to be sex-biased; thus, we have delved into the genetic and cellular disease mechanisms to better understand how sex differences in these pathways may result in sex differences in brain structure and function.

Rett syndrome is the most evident neurodevelopmental phenotype identified in the pathway analysis, which found that female-biased miRNAs were enriched for that disorder compared to male-biased miRNAs. Caused by loss-of-function mutations in X-linked MeCp2, Rett syndrome is almost exclusively diagnosed in females, as a mutation in the hemizygous male results in lethality. Normally, MeCp2 functions throughout the genome by reading the DNA methylation status and recruiting other chromatin modifiers to repress gene expression. When this function is lost in patients with Rett syndrome, global epigenetic changes result in gene expression changes, which alter the development of structures in the developing brain to ultimately drive a phenotype of cognitive disability [75]. Other genetic variations in MeCp2 are linked to various NDDs, and the phenotype of Rett syndrome shows considerable overlap with other NDD phenotypes, suggesting some shared etiology.

The disruption of miRNA expression downstream of the MeCp2 mutation has been previously documented [76], and further studies have demonstrated specific consequences on pathways and processes in neurodevelopment due to dysregulation of Rett pathway miRNAs [77, 78]. Therefore, identifying Rett syndrome-associated miRNAs among our sex-biased miRNAs suggests that normal neurodevelopmental functions performed by these miRNAs occur in a sex-biased manner in healthy mice. Furthermore, studies have shown female-biased expression of MeCp2 in the immediate postnatal period in rodents, a trend that appears to be driven by dimorphic sex hormone production during this sensitive period of brain development [79]. Not only does this research conclude that sex-biased MeCp2 expression canalizes sex differences in behavior, but it also provides a plausible explanation for the sex differences observed in the expression of miRNAs in the Rett syndrome pathway during normal neurodevelopment.

Two neurodegenerative disease pathways, Alzheimer’s disease (AD) and Huntington’s disease (HD), were enriched in multiple pathway analyses of sex-biased genes and miRNAs. Although it may be expected that AD and HD pathways are enriched in aged brains rather than in the embryonic tissue used here, many key components of these pathways function in normal neurodevelopment. For example, Notch signaling through the AD pathway is vital for growth cone guidance and reelin for cortical neuron migration [79], while the Huntington gene (*Htt*) has pleiotropic roles in coordinating differentiation via epigenetic mechanisms [80]. Therefore, our results indicate sex differences in these normal neurodevelopmental processes. Second, there is considerable research interest in miRNAs and their contributions to AD and HD pathogenesis. Several miRNAs have been demonstrated to differ between cases and controls for each disease, but the species and age differences between our findings prevent speculation linking female-biased *miR-9-3p* expression in the embryonic mouse brain to increased expression of miR-9-3p in the brains of patients with AD, where AD is more prevalent in females.

However, the enrichment of neurodegenerative disease pathways in our dataset is reminiscent of the neurodevelopmental hypothesis of neurodegenerative diseases. This hypothesis posits that disease etiology is in part due to structural and functional aberrations that arise as the brain develops. Although the brain can initially compensate for lost function, environmental and/or age-related changes may reveal these deficits. Thus, although the disease phenotype is expressed later in life, the mechanistic origins of the disease begin during neurodevelopment [80, 81]. This hypothesis is supported by various lines of evidence, including the essential functions of the AD and HD pathway genes in embryonic neurodevelopment. Therefore, our data conform to this hypothesis by reiterating the importance of neurodegenerative disease pathways in development but with an additional dimension of sex. We showed that these pathways are shared among sex-biased genes and miRNAs. This trend could not only contribute to distinct sex differences in neurodevelopmental processes, as discussed above, but it could also drive sex differences in a pathological context where developmental aberrations in sex-biased pathways could result in sex differences in disease outcomes later in life.

### Sex-biased miRNAs associated with neurodevelopmental disorders

Sex-biased miRNAs of interest have been frequently described in the context of NDDs, and pathway analysis has revealed several promising disease categories enriched among sex-biased miRNAs and their target genes (Fig. 5A–F). In addition, we used SNP-mining approaches to determine whether these miRNAs are nearby SNPs associated with clinically relevant phenotypes. The results indicated that MIR9 and MIR206 reside in regions containing many SNPs related to neurological traits according to the database and literature-based inquiries (Fig. 6A–C).

Additionally, we considered whether any of the identified SNPs could affect miRNA function or expression. None of the SNPs were within the miRNA genes themselves but in the surrounding intergenic regions. A few SNPs identified could potentially affect the genomic elements that regulate miR-9 expression. Two SNPs downstream of MIR9-3, rs176644 and rs176647, each reside within distal enhancer-like signatures (ENCODE) with TF binding and CTCF binding sites (ORegAnno). The latter also strongly interacted with the MIR9-3 TSS (GeneHancer) (Additional file 1: Fig. S5). This combination of features suggests possible and likely enhancer functions for the respective SNPs regarding MIR9-3, providing a plausible functional basis for their associated trait, insomnia. Insomnia is considered a neurological disorder that shares considerable comorbidity and genetic basis with other neuropsychiatric disorders [82].

In addition to the direct consequences of two insomnia-associated SNPs, we suggest indirect impacts on *miR-9-5p/3p* function due to several SNPs near MIR9-2. Thirteen neurologically associated SNPs lie downstream of MIR9-2, with the non-coding region of a lncRNA that shares a TSS with MIR9-2 (Additional file 1: Fig. S5). LINC00461 is not only co-transcribed with MIR9-2, but is also known to act in a regulatory loop together with *miR-9-5p/3p* in neurological contexts [57, 83]. Thus, intronic SNPs that may alter the splicing of LINC00461 could alter the regulation of miR-9 by LINC00461, which may subsequently alter *miR-9-5p/3p* levels in the brain. Evidence that SNPs associated with neurological disorders may alter the regulation of brain-enriched *miR-9-5p/3p* provides compelling reasons to further investigate the role of these miRNAs in normal and aberrant brain function.

### Integrating sex-biased mRNA and miRNA expression

Having generated data with both RNA-seq and small RNA-seq methods, we had resources to consider how sex-biased miRNA expression may impact sex-biased mRNA levels. Using a set of stringently verified miRNA:target interactions, we identified eight pairs in which both miRNA and its target mRNA demonstrated sex-biased expression in the E15.5 mouse brain. Six of the eight miRNA:target pairs showed discordant expression between the sexes, where miRNA was expressed more highly in one sex and its mRNA target was expressed more strongly in the other. This pattern indicates that high miRNA expression reduces the level of the target mRNA, consistent with miRNA-induced mRNA degradation [84]. The remaining two miRNA:target pairs showed concordant expression between the sexes, whereas the miRNA and its target were highly expressed in one sex.

Alternatively, we can consider these results assuming that miRNA-induced repression occurs via translational inhibition rather than via mRNA degradation [85]. This scenario could result in buffering of sex differences at the protein level for the two concordant pairs; for example, higher expression of *Ddit4* mRNA in males than in females could be compensated for by higher *miR-221-3p* expression in males, as this miRNA targets *Ddit4* mRNA and could reduce the level of DDIT4 protein production to make it more similar to that in females. The normalization of protein levels by miRNAs when target mRNA levels vary is a function that has evolved to ensure phenotypic stability and to minimize the effect of environmental variation [86, 87]. The idea that miRNAs may buffer sex differences in mRNA expression is consistent with the idea of compensation, an evolutionary perspective that has been increasingly used to frame sex differences in the brain. The premise for compensation is that while sexual dimorphic factors have sculpted differences in the brain, not all those differences benefit; therefore, evolutionary forces have emerged to mitigate sex differences [88]. However, to test whether miRNAs buffer sex differences in the developing mouse brain, we need to add protein data to the equation for miRNA:target pairs such as miR-221-3p:Ddit4.

## Limitations and future directions

This study lacks the integration of proteomics into the transcriptome and miRnome data. miRNAs function by either degrading mRNA or repressing the translation of mRNA into protein [89]. Therefore, correlating miRNA expression with the expression levels of their predicted target mRNAs can reveal the portion of interactions captured by the former mechanism. However, without the protein component, the latter could not be detected. Ultimately, miRNA action represses protein levels and contributes to the uncoupling of mRNA levels at the protein level. Including mass spectrometry experiments to investigate the proteomics of the developing brain would yield results that demonstrate more clearly the consequences of sex differences in gene expression in the developing brain and how miRNAs contribute to sex differences at the protein endpoint.

A key limitation of RNA-seq and small RNA-seq, as well as all downstream analyses, is the use of whole brain tissue and bulk RNA-seq. Sex-based differences were evident in the brain. However, sex-differential RNA expression can be specific to a brain region, cell type, or even a particular subcellular location. Unsurprisingly, a tissue as heterogeneous as the brain shows highly specific spatial expression patterns, arguing that sequencing should be performed on a more specific region of the brain rather than using the whole brain. Furthermore, by using the entire brain, the analysis can only show a composite trend; only those sex biases consistent across the whole brain can be detected. While these are the sex biases that exert the largest effect on the brain, key spatial variations in sex differences will be masked [89]. It is vital to be aware that our RNA-seq and small RNA-seq data only capture sex differences at the whole-brain level.

The ideal strategy to overcome the limitations inherent in studying the highly heterogeneous brain is to use high-throughput methodology with the ability to capture spatial information, namely single-cell RNA-seq (scRNA-seq) and/or spatial transcriptomics. In contrast to the bulk RNA-seq methods used in our study, scRNA-seq resolves gene expression in individual cells, which confers a huge advantage when studying diverse neurological cell types [89]. These advantages are also apparent in spatial transcriptomics: sequencing libraries constructed mapped to sectioned tissue enable the integration of gene expression data with positional information to generate expression atlases with a resolution near that of scRNA-seq [90]. Both techniques have become increasingly refined and cost accessible in recent years, with scRNA-seq being adapted to work with small RNAs [91], with the potential to generate robust datasets that could enable us to obtain a more nuanced understanding of sex-biased RNAs in the brain, incorporating the spatial dimension of expression.

Interlinked, with the idea of dynamic spatial expression, is temporal expression. E15.5 was selected to study sex-biased expression in the brain because it is 4 days after gonadal sex determination; therefore, both sex chromosomes and sex hormones may exert an effect on gene expression at this time. At E15.5, embryonic testes begin to generate T, while at the same time, the highly plastic embryonic brain is receptive to the influence of T through sex steroid hormone receptors [92–96]. Aromatase expression is also present as early as E9.5, by E16.6 over 400 neurons are positive for aromatase expression [97]. T production is possible from ~ E12.0 [98], reaches a peak at ~ E17-18, followed by a rapid decline just prior to birth and then a spike in production in the first 24 h [99, 100]. Therefore, we might expect a different set of differentially expressed mRNA and miRNAs had we additionally investigated more timepoints (before and after E15.5). A larger study design could include several different stages of development, not only to identify the window for maximal sexual differentiation of the brain, but also to track differences in the rate of brain development, which has previously been demonstrated in humans but not in mice [72].

A final consideration when interpreting the findings presented here was the use of a mouse model of neurodevelopment. Not only are they a tractable laboratory species that has been extensively characterized, but, in general, mice share the core tenets of this study with humans, including mechanisms of sex determination and sexual differentiation, the general blueprint of brain development, and ~ 90% of their genome [101–103]. However, nuanced species differences in each of these elements limit their human applicability. In particular, the dynamic evolution of non-coding transcripts, including miRNAs [104] and human NDDs, is thought to arise predominantly in aspects of the brain that are unique to humans and therefore cannot be recapitulated in rodent models [105]. Finally, mice cannot be used to probe the effect of gender on NDDs. Highly entangled biological sex and gender differences have been proposed to modify the sex bias observed in various NDDs [7, 9]. As they lack the unique human experience of gender, mouse models can only be used to investigate the effects of biological sex [106].

## Perspectives and significance

This article demonstrates robust sex differences for a portion of genes and miRNAs expressed in the embryonic mouse brain, suggesting that several candidate RNAs whose role in establishing sex differences in the brain may be further investigated. Although sex-differential expression patterns have previously been explored in the brain, this is the first study to use RNA-seq in parallel with small RNA-seq to gain new insight into how sex differences in miRNA and mRNA targets interact. Furthermore, gene ontology analysis suggested essential functions for sex-biased genes and miRNAs in known neurodevelopmental pathways. These pathways represent another avenue through which we can explore how sex differences arise in the developing brain. Together, these findings reinforce the importance of cataloguing sex differences in molecular biology research and highlight miRNAs and pathways of interest that may be important for sexual differentiation in the mouse and possibly the human brain.

## Conclusions

Here, we characterized sex differences in the developing mouse brain at the RNA level. Sex-biased genes, miRNAs, and the pathways in which they act greatly affect neurodevelopmental processes. These findings demonstrate the link between sex-biased genes and miRNA expression and their consequences on neurodevelopment, emphasizing the importance of RNA in establishing sex differences in the prenatal mouse brain. An improved understanding of which miRNAs contribute to sex differences in the developing mouse brain prompts us to ask how and why these sex-biased RNA expression patterns arise.

### Supplementary Information

**Additional file 1: Table S1*.*** RT-qPCR primer sequences. **Table S2.** miRNA RT-qPCR primer sequences. **Table S3.** Top 10 miRNAs in small RNA-seq data by combined (n = 6) read count. **Figure S1.** RNA-seq mapping statistics and read quality. **A** Read count data for each sequenced sample in raw file, in the output from trimming and QC, and following mapping to the mm9 reference genome for both forward and reverse sequences. **B** MultiQC plot of aggregated phred scores for all samples across the 125 bp read length. **Figure S2.** Small RNA-seq mapping statistics and read quality. **A** Read count data for each sequenced sample in the raw file, in the output from trimming and QC, and following mapping to the mm9 reference genome, with the percentage of trimmed reads successfully mapped in brackets. **B** FastQC plot of a representative sample showing phred score across the 50 bp untrimmed read. **C** Read length distribution plot for a representative sample showing that read lengths peak at ~ 22 bp. Figure S4. Conservation of sex-biased miRNAs of interest between mouse and human genomes. Genome browser screenshots depict mouse miRNAs (black) mapping to the UCSC hg38 genome (green). “Cons 100 Verts” indicates PhyloP scores across 100 vertebrates and the “Multiz alignment” track shows human and mouse sequences at base pair resolution. Sequences with 100% conservation are highlighted yellow. All paralogs have been included for the 7 miRNAs of interest: a) miR-9-3p, b) miR-10b-5p, c) miR-101-3p, d) miR-199-5p, e) miR-200-3p, f) miR-205-5p, g) miR-206-3p. **Figure S5.** Genome browser screenshot from hg38 assembly showing the MIR9-2 and MIR9-3 loci, respectively. Yellow highlighting indicates the miRNA gene, pink indicates neurologically associated SNPs, and green is the TSS for each miRNA gene.

**Additional file 2: Data S1*.*** Excel file providing the complete RNA-seq and Gene ontology analysis results. Data is separated into sheets by sex (Purple for Female analyses; Green for Male analyses).

**Additional file 3: Data S2*.*** Excel file providing the complete small RNA-seq and Gene ontology analysis results. Data is separated into sheets by sex (Purple for Female analyses; Green for Male analyses).

**Additional file 4: Data S3*.*** List of miRNAs, associated SNP/variant ID and phenotype.

## Data Availability

Raw sequencing data for RNA-seq and small RNA-seq experiments are available at GSE211816 in the NCBI GEO database.

## Abbreviations

miRNA: MicroRNA
RNA-seq: RNA-sequencing
small RNA-seq: Small RNA-sequencing
NDD: Neurodevelopmental disorder

## References

[1] Beery AK, Zucker I (2011). Sex bias in neuroscience and biomedical research. Neurosci Biobehav Rev.

[2] McCarthy MM, Arnold AP, Ball GF, Blaustein JD, De Vries GJ (2012). Sex differences in the brain: the not so inconvenient truth. J Neurosci.

[3] Shansky RM (2019). Are hormones a “female problem” for animal research? Outdated gender stereotypes are influencing experimental design in laboratory animals. Science.

[4] Middeldorp CM, Hammerschlag AR, Ouwens KG, Groen-Blokhuis MM, St. Pourcain B, Greven CU, Pappa I, Tiesler CMT, Ang W, Nolte IM (2016). A genome-wide association meta-analysis of attention-deficit/hyperactivity disorder symptoms in population-based pediatric cohorts. J Am Acad Child Adolesc Psychiatry.

[5] Henriksen MG, Nordgaard J, Jansson LB (2017). Genetics of schizophrenia: overview of methods, findings and limitations. Front Hum Neurosci.

[6] Courchesne E, Mouton PR, Calhoun ME, Semendeferi K, Ahrens-Barbeau C, Hallet MJ, Barnes CC, Pierce K (2011). Neuron number and size in prefrontal cortex of children with autism. JAMA.

[7] Lynch A, Davison K (2022). Gendered expectations on the recognition of ADHD in young women and educational implications. Irish Educ Stud.

[8] Santos S, Ferreira H, Martins J, Gonçalves J, Castelo-Branco M (2022). Male sex bias in early and late onset neurodevelopmental disorders: shared aspects and differences in autism spectrum disorder, attention deficit/hyperactivity disorder, and schizophrenia. Neurosci Biobehav Rev.

[9] Moore I, Morgan G, Welham A, Russell G (2022). The intersection of autism and gender in the negotiation of identity: a systematic review and metasynthesis. Fem Psychol.

[10] Arnold AP (2017). A general theory of sexual differentiation. J Neurosci Res.

[11] Chini M, Hanganu-Opatz IL (2021). Prefrontal cortex development in health and disease: lessons from rodents and humans. Trends Neurosci.

[12] Qureshi IA, Mehler MF (2012). Emerging roles of non-coding RNAs in brain evolution, development, plasticity and disease. Nat Rev Neurosci.

[13] Bartel DP (2018). Metazoan microRNAs. Cell.

[14] Ohtsuka M, Ling H, Doki Y, Mori M, Calin GA (2015). MicroRNA processing and human cancer. J Clin Med.

[15] Friedman RC, Farh KKH, Burge CB, Bartel DP (2009). Most mammalian mRNAs are conserved targets of microRNAs. Genome Res.

[16] Sun E, Shi Y (2014). MicroRNAs: small molecules with big roles in neurodevelopment and diseases. Exp Neurol.

[17] Geaghan M, Cairns MJ (2015). MicroRNA and posttranscriptional dysregulation in psychiatry. Biol Psychiatry.

[18] McCarthy MM, Pickett LA, VanRyzin JW, Kight KE (2015). Surprising origins of sex differences in the brain. Horm Behav.

[19] Ingleby FC, Flis I, Morrow EH (2015). Sex-biased gene expression and sexual conflict throughout development. Cold Spring Harb Perspect Biol.

[20] Koturbash I, Zemp F, Kolb B, Kovalchuk O (2011). Sex-specific radiation-induced microRNAome responses in the hippocampus, cerebellum and frontal cortex in a mouse model. Mutat Res Genetic Toxicol Environ Mutagenes.

[21] Cui C, Yang W, Shi J, Zhou Y, Yang J, Cui Q, Zhou Y (2018). Identification and analysis of human sex-biased microRNAs. Genom Proteom Bioinform.

[22] Hirsch MM, Brusco J, Vaccaro T, Margis R, Moreira JE, Gottfried C, Rasia-Filho AA (2018). Sex differences and estrous cycle changes in synaptic plasticity-related microRNA in the rat medial amygdala. Neuroscience.

[23] Kodama L, Guzman E, Etchegaray JI, Li Y, Sayed FA, Zhou L, Zhou Y, Zhan L, Le D, Udeochu JC (2020). Microglial microRNAs mediate sex-specific responses to tau pathology. Nat Neurosci.

[24] Corrales WA, Silva JP, Parra CS, Olave FA, Aguayo FI, Román-Albasini L, Aliaga E, Venegas-Zamora L, Avalos AM, Rojas PS (2021). Sex-dependent changes of miRNA levels in the hippocampus of adrenalectomized rats following acute corticosterone administration. ACS Chem Neurosci.

[25] Luo PX, Manning CE, Fass JN, Williams VA, Hao R, Campi KL, Trainor BC (2021). Sex-specific effects of social defeat stress on miRNA expression in the anterior BNST. Behav Brain Res.

[26] Mavrikaki M, Pantano L, Potter D, Rogers-Grazado MA, Anastasiadou E, Slack FJ, Amr SS, Ressler KJ, Daskalakis NP, Chartoff E (2019). Sex-dependent changes in miRNA expression in the bed nucleus of the stria terminalis following stress. Front Mol Neurosci.

[27] McKibben LA, Dwivedi Y (2021). Early-life stress induces genome-wide sex-dependent miRNA expression and correlation across limbic brain areas in rats. Epigenomics.

[28] Ziats MN, Rennert OM (2014). Identification of differentially expressed microRNAs across the developing human brain. Mol Psychiatry.

[29] Kim CK, Pak TR (2020). miRNA degradation in the mammalian brain. Am J Phys Cell Physiol.

[30] Morgan CP, Bale TL (2011). Early prenatal stress epigenetically programs dysmasculinization in second-generation offspring via the paternal lineage. J Neurosci.

[31] Morgan CP, Bale TL (2017). Sex differences in microRNA-mRNA networks: examination of novel epigenetic programming mechanisms in the sexually dimorphic neonatal hypothalamus. Biol Sex Differ.

[32] Murphy SJ, Lusardi TA, Phillips JI, Saugstad JA (2014). Sex differences in microRNA expression during development in rat cortex. Neurochem Int.

[33] Arnold AP, Chen X (2009). What does the “four core genotypes” mouse model tell us about sex differences in the brain and other tissues?. Front Neuroendocrinol.

[34] McCarthy MM (2020). A new view of sexual differentiation of mammalian brain. J Comp Physiol A Neuroethol Sens Neural Behav Physiol.

[35] Afgan E, Sloggett C, Goonasekera N, Makunin I, Benson D, Crowe M, Gladman S, Kowsar Y, Pheasant M, Horst R (2015). Genomics virtual laboratory: a practical bioinformatics workbench for the cloud. PLoS ONE.

[36] Bolger AM, Lohse M, Usadel B (2014). Trimmomatic: a flexible trimmer for Illumina sequence data. Bioinformatics.

[37] Andrews S. FastQC—a quality control tool for high throughput sequence data. In: Babraham bioinformatics. 2010.

[38] Kim D, Pertea G, Trapnell C, Pimentel H, Kelley R, Salzberg SL (2013). TopHat2: accurate alignment of transcriptomes in the presence of insertions, deletions and gene fusions. Genome Biol.

[39] Trapnell C, Williams BA, Pertea G, Mortazavi A, Kwan G, Van Baren MJ, Salzberg SL, Wold BJ, Pachter L (2010). Transcript assembly and quantification by RNA-Seq reveals unannotated transcripts and isoform switching during cell differentiation. Nat Biotechnol.

[40] Liao Y, Smyth GK, Shi W (2014). featureCounts: an efficient general purpose program for assigning sequence reads to genomic features. Bioinformatics.

[41] Love MI, Huber W, Anders S (2014). Moderated estimation of fold change and dispersion for RNA-seq data with DESeq2. Genome Biol.

[42] Risso D, Ngai J, Speed TP, Dudoit S (2014). Normalization of RNA-seq data using factor analysis of control genes or samples. Nat Biotechnol.

[43] Friedländer MR, MacKowiak SD, Li N, Chen W, Rajewsky N (2012). miRDeep2 accurately identifies known and hundreds of novel microRNA genes in seven animal clades. Nucleic Acids Res.

[44] Martin M (2011). Cutadapt removes adapter sequences from high-throughput sequencing reads. EMBnetjournal.

[45] Langmead B, Salzberg SL (2012). Fast gapped-read alignment with Bowtie 2. Nat Methods.

[46] Hauberg ME, Roussos P, Grove J, Børglum AD, Mattheisen M (2016). Analyzing the role of microRNAs in schizophrenia in the context of common genetic risk variants. JAMA Psychiat.

[47] Ge SX, Jung D, Jung D, Yao R (2020). ShinyGO: a graphical gene-set enrichment tool for animals and plants. Bioinformatics.

[48] Kern F, Fehlmann T, Solomon J, Schwed L, Grammes N, Backes C, van Keuren-Jensen K, Craig DW, Meese E, Keller A (2021). miEAA 2.0: integrating multi-species microRNA enrichment analysis and workflow management systems. Nucleic Acids Res.

[49] Ning L, Cui T, Zheng B, Wang N, Luo J, Yang B, Du M, Cheng J, Dou Y, Wang D (2021). MNDR v3.0: mammal ncRNA–disease repository with increased coverage and annotation. Nucleic Acids Res.

[50] Kent WJ (2002). BLAT—the BLAST-like alignment tool. Genome Res.

[51] Cariaso M, Lennon G (2012). SNPedia: a wiki supporting personal genome annotation, interpretation and analysis. Nucleic Acids Res.

[52] MacArthur J, Bowler E, Cerezo M, Gil L, Hall P, Hastings E, Junkins H, McMahon A, Milano A, Morales J (2017). The new NHGRI-EBI Catalog of published genome-wide association studies (GWAS catalog). Nucleic Acids Res.

[53] Sinnott-Armstrong N, Naqvi S, Rivas M, Pritchard JK (2021). Gwas of three molecular traits highlights core genes and pathways alongside a highly polygenic background. Elife.

[54] Enright AJ, John B, Gaul U, et al. MicroRNA targets in Drosophila. Genome Biol. 2003;5(1):R1. 10.1186/gb-2003-5-1-r1. 10.1186/gb-2003-5-1-r1 PMC395733 14709173

[55] McCarthy MM, Nugent BM, Lenz KM (2017). Neuroimmunology and neuroepigenetics in the establishment of sex differences in the brain. Nat Rev Neurosci.

[56] Shibata M, Nakao H, Kiyonari H, Abe T, Aizawa S (2011). MicroRNA-9 regulates neurogenesis in mouse telencephalon by targeting multiple transcription factors. J Neurosci.

[57] Tovo-Rodrigues L, Quinte GC, Brum CB, Ghisleni G, Bastos CR, Oliveira DIO, Barros FC, Barros AJD, Santos IS, Rohde LA (2019). The role of MIR9-2 in shared susceptibility of psychiatric disorders during childhood: a population-based birth cohort study. Genes.

[58] Hansen T, Olsen L, Lindow M, Jakobsen KD, Ullum H, Jonsson E, Andreassen OA, Djurovic S, Melle I, Agartz I (2007). Brain expressed microRNAs implicated in schizophrenia etiology. PLoS ONE.

[59] Toma C, Torrico B, Hervás A, Salgado M, Rueda I, Valdés-Mas R, Buitelaar JK, Rommelse N, Franke B, Freitag C (2015). Common and rare variants of microRNA genes in autism spectrum disorders. World J Biol Psychiatry.

[60] Bramble MS, Roach L, Lipson A, Vashist N, Eskin A, Ngun T, Gosschalk JE, Klein S, Barseghyan H, Arboleda VA (2016). Sex-specific effects of testosterone on the sexually dimorphic transcriptome and epigenome of embryonic neural stem/progenitor cells. Sci Rep.

[61] Dewing P, Shi T, Horvath S, Vilain E (2003). Sexually dimorphic gene expression in mouse brain precedes gonadal differentiation. Mol Brain Res.

[62] Shi L, Zhang Z, Su B (2016). Sex biased gene expression profiling of human brains at major developmental stages. Sci Rep.

[63] Trabzuni D, Ramasamy A, Imran S, Walker R, Smith C, Weale ME, Hardy J, Ryten M (2013). Widespread sex differences in gene expression and splicing in the adult human brain. Nat Commun.

[64] Yang X, Schadt EE, Wang S, Wang H, Arnold AP, Ingram-Drake L, Drake TA, Lusis AJ (2006). Tissue-specific expression and regulation of sexually dimorphic genes in mice. Genome Res.

[65] Jonsson ME, Nelander Wahlestedt J, Akerblom M, Kirkeby A, Malmevik J, Brattaas PL, Jakobsson J, Parmar M (2015). Comprehensive analysis of microRNA expression in regionalized human neural progenitor cells reveals microRNA-10 as a caudalizing factor. Development.

[66] Roese-Koerner B, Stappert L, Berger T, Braun NC, Veltel M, Jungverdorben J, Evert BO, Peitz M, Borghese L, Brustle O (2016). Reciprocal regulation between bifunctional miR-9/9(*) and its transcriptional modulator notch in human neural stem cell self-renewal and differentiation. Stem Cell Rep.

[67] Morante J, Vallejo DM, Desplan C, Dominguez M (2013). Conserved miR-8/miR-200 defines a glial niche that controls neuroepithelial expansion and neuroblast transition. Dev Cell.

[68] Lippi G, Fernandes CC, Ewell LA, John D, Romoli B, Curia G, Taylor SR, Frady EP, Jensen AB, Liu JC (2016). MicroRNA-101 regulates multiple developmental programs to constrain excitation in adult neural networks. Neuron.

[69] Mellios N, Feldman DA, Sheridan SD, Ip JPK, Kwok S, Amoah SK, Rosen B, Rodriguez BA, Crawford B, Swaminathan R (2018). MeCP2-regulated miRNAs control early human neurogenesis through differential effects on ERK and AKT signaling. Mol Psychiatry.

[70] Lobentanzer S, Hanin G, Klein J, Soreq H (2019). Integrative transcriptomics reveals sexually dimorphic control of the cholinergic/neurokine interface in schizophrenia and bipolar disorder. Cell Rep.

[71] Evans AC (2006). The NIH MRI study of normal brain development. Neuroimage.

[72] Lenroot RK, Gogtay N, Greenstein DK, Wells EM, Wallace GL, Clasen LS, Blumenthal JD, Lerch J, Zijdenbos AP, Evans AC (2007). Sexual dimorphism of brain developmental trajectories during childhood and adolescence. Neuroimage.

[73] Hanamsagar R, Alter MD, Block CS, Sullivan H, Bolton JL, Bilbo SD (2017). Generation of a microglial developmental index in mice and in humans reveals a sex difference in maturation and immune reactivity. Glia.

[74] Geary DC (2018). Evolutionary perspective on sex differences in the expression of neurological diseases. Prog Neurobiol.

[75] Good VK, Vincent JB, Ausió J (2021). MeCP2: the genetic driver of rett syndrome epigenetics. Front Genet.

[76] Urdinguio RG, Fernandez AF, Lopez-Nieva P, Rossi S, Huertas D, Kulis M, Liu CG, Croce C, Calin GA, Esteller M (2010). Disrupted microRNA expression caused by Mecp2 loss in a mouse model of Rett syndrome. Epigenetics.

[77] Cheng TL, Wang Z, Liao Q, Zhu Y, Zhou WH, Xu W, Qiu Z (2014). MeCP2 suppresses nuclear microRNA processing and dendritic growth by regulating the DGCR8/Drosha complex. Dev Cell.

[78] Kurian JR, Bychowski ME, Forbes-Lorman RM, Auger CJ, Auger AP (2008). Mecp2 organizes juvenile social behavior in a sex-specific manner. J Neurosci.

[79] Bothwell M, Giniger E (2000). Minireview Alzheimer’s disease: neurodevelopment converges with neurodegeneration. Cell.

[80] Kerschbamer E, Biagioli M (2016). Huntington's disease as neurodevelopmental disorder: altered chromatin regulation, coding, and non-coding RNA transcription. Front Neurosci.

[81] Van Der Plas E, Schultz JL, Nopoulos PC (2020). The neurodevelopmental hypothesis of Huntington’s disease. J Huntington’s Dis.

[82] Jansen PR, Watanabe K, Stringer S, Skene N, Bryois J, Hammerschlag AR, de Leeuw CA, Benjamins JS, Muñoz-Manchado AB, Nagel M (2019). Genome-wide analysis of insomnia in 1,331,010 individuals identifies new risk loci and functional pathways. Nat Genet.

[83] Pinson MR, Miranda RC (2019). Noncoding RNAs in development and teratology, with focus on effects of cannabis, cocaine, nicotine, and ethanol. Birth Defects Res.

[84] Nilsen TW (2007). Mechanisms of microRNA-mediated gene regulation in animal cells. Trends Genet.

[85] Carthew RW, Sontheimer EJ (2009). Origins and mechanisms of miRNAs and siRNAs. Cell.

[86] Strovas TJ, Rosenberg AB, Kuypers BE, Muscat RA, Seelig G (2014). MicroRNA-based single-gene circuits buffer protein synthesis rates against perturbations. ACS Synth Biol.

[87] Posadas DM, Carthew RW (2014). MicroRNAs and their roles in developmental canalization. Curr Opin Genet Dev.

[88] De Vries GJ (2004). Minireview: sex differences in adult and developing brains: compensation, compensation, compensation. Endocrinology.

[89] Lu T, Mar JC (2020). Investigating transcriptome-wide sex dimorphism by multi-level analysis of single-cell RNA sequencing data in ten mouse cell types. Biol Sex Differ.

[90] Burgess DJ (2019). Spatial transcriptomics coming of age. Nat Rev Genet.

[91] Liu W, Shomron N (2021). Analysis of microRNA regulation in single cells. Methods Mol Biol.

[92] Dehorter N, Del Pino I (2020). Shifting developmental trajectories during critical periods of brain formation. Front Cell Neurosci.

[93] Young WJ, Chang C (1998). Ontogeny and autoregulation of androgen receptor mRNA expression in the nervous system. Endocrine.

[94] MacLusky NJ, Walters MJ, Clark AS, Toran-Allerand CD (1994). Aromatase in the cerebral cortex, hippocampus, and mid-brain: ontogeny and developmental implications. Mol Cell Neurosci.

[95] Yilmaz MB, Zhao H, Brooks DC, Fenkci IV, Imir-Yenicesu G, Attar E, Akbal E, Kaynak BA, Bulun SE (2015). Estrogen receptor alpha (Esr1) regulates aromatase (Cyp19a1) expression in the mouse brain. Neuro Endocrinol Lett.

[96] Fan X, Warner M, Gustafsson JA (2006). Estrogen receptor beta expression in the embryonic brain regulates development of calretinin-immunoreactive GABAergic interneurons. Proc Natl Acad Sci USA.

[97] Wartenberg P, Farkas I, Csillag V, Colledge WH, Hrabovszky E, Boehm U (2021). Sexually dimorphic neurosteroid synthesis regulates neuronal activity in the murine brain. J Neurosci.

[98] Clarkson J, Herbison AE (2016). Hypothalamic control of the male neonatal testosterone surge. Philos Trans R Soc Lond B Biol Sci.

[99] O'Shaughnessy PJ, Baker P, Sohnius U, Haavisto AM, Charlton HM, Huhtaniemi I (1998). Fetal development of Leydig cell activity in the mouse is independent of pituitary gonadotroph function. Endocrinology.

[100] Ward IL, Ward OB, Affuso JD, Long WD, French JA, Hendricks SE (2003). Fetal testosterone surge: specific modulations induced in male rats by maternal stress and/or alcohol consumption. Horm Behav.

[101] Zhao X, Bhattacharyya A (2018). Human models are needed for studying human neurodevelopmental disorders. Am J Hum Genet.

[102] Marshall Graves JA, Shetty S (2001). Sex from W to Z: evolution of vertebrate sex chromosomes and sex determining genes. J Exp Zool.

[103] Waterston RH, Lindblad-Toh K, Birney E, Rogers J, Abril JF, Agarwal P, Agarwala R, Ainscough R, Alexandersson M, An P (2002). Initial sequencing and comparative analysis of the mouse genome. Nature.

[104] Somel M, Liu X, Tang L, Yan Z, Hu H, Guo S, Jiang X, Zhang X, Xu G, Xie G (2011). MicroRNA-driven developmental remodeling in the brain distinguishes humans from other primates. PLoS Biol.

[105] Silbereis JC, Pochareddy S, Zhu Y, Li M, Sestan N (2016). The cellular and molecular landscapes of the developing human central nervous system. Neuron.

[106] Ritz SA, Greaves L (2022). Transcending the male–female binary in biomedical research: constellations, heterogeneity, and mechanism when considering sex and gender. Int J Environ Res Public Health.

