# Supplementary material for: Sex-biased gene and microRNA expression in the developing mouse brain is associated with neurodevelopmental functions and neurological phenotypes
Source: Biol Sex Differ. 2023 Sep 7;14:57. doi: 10.1186/s13293-023-00538-3 (PMC10486049; doi:10.1186/s13293-023-00538-3)
Supplement: Supplementary file 1 — Additional file 1: Table S1. RT-qPCR primer sequences. Table S2. miRNA RT-qPCR primer sequences. Table S3. Top 10 miRNAs in small RNA-seq data by combined (n = 6) read count. Figure S1. RNA-seq mapping statistics and read quality. A Read count data for each sequenced sample in raw file, in the output from trimming and QC, and following mapping to the mm9 reference genome for both forward and reverse sequences. B MultiQC plot of aggregated phred scores for all samples across the 125 bp read length. Figure S2. Small RNA-seq mapping statistics and read quality. A Read count data for each sequenced sample in the raw file, in the output from trimming and QC, and following mapping to the mm9 reference genome, with the percentage of trimmed reads successfully mapped in brackets. B FastQC plot of a representative sample showing phred score across the 50 bp untrimmed read. C Read length distribution plot for a representative sample showing that read lengths peak at ~ 22 bp. Figure S4. Conservation of sex-biased miRNAs of interest between mouse and human genomes. Genome browser screenshots depict mouse miRNAs (black) mapping to the UCSC hg38 genome (green). “Cons 100 Verts” indicates PhyloP scores across 100 vertebrates and the “Multiz alignment” track shows human and mouse sequences at base pair resolution. Sequences with 100% conservation are highlighted yellow. All paralogs have been included for the 7 miRNAs of interest: a) miR-9-3p, b) miR-10b-5p, c) miR-101-3p, d) miR-199-5p, e) miR-200-3p, f) miR-205-5p, g) miR-206-3p. Figure S5. Genome browser screenshot from hg38 assembly showing the MIR9-2 and MIR9-3 loci, respectively. Yellow highlighting indicates the miRNA gene, pink indicates neurologically associated SNPs, and green is the TSS for each miRNA gene. [file 13293_2023_538_MOESM1_ESM.docx]

**Additional Tables/figures**

Table S1: RT-qPCR primer sequences.

| **Gene (RefSeq mRNA transcript ID)** | **Oligonucleotide primer sequence (5’-3’)** | | **Primer efficiency (%)** |
| --- | --- | --- | --- |
|  | **Forward** | **Reverse** |  |
| *ActB* (NM_007393) | GGCTGTATTCCCCTCCATCG | CCAGTTGGTAATGCCATGT | 90% |
| *Aff2* (NM_008032) | CTACCACAACCACTACCACTA | CCAGGTGACTGCTATCCATAA | 168% |
| *Asxl3* (NM_001167777) | CTGCTATTCCAGCGTCAGATAC | GCCTCAACTGTTCCTCTCATAC | 73% |
| *Bmpr2* (NM_007561) | GGCACATAGGTCCCAAGAAA | CTGTTGCTCTCGTCTCAGTAAA | 76% |
| *Cecr5* (NM_144815) | CTCAGGAGAAGGGATGGATTTC | CACTGAGGCACAGAGGTAAAG | 112% |
| *Chmp1a* (NM_145606) | GAGAGTAGCTGCAGTCTCGG | CCAGCTCCCCATCCCTACTA | 104% |
| *Col9a1* (NM_007740) | GATTGGCGTGGAAAGAACAAG | CAAAGCCATCCGCATCAATC | 92% |
| *Dicer1* (NM_148948) | CTCGAGATCCTGCTGCGCAAATA | GAATCACTCCAGGACACGTAA | 137% |
| *Eif2s3y* (NM_012011) | TGCTCCAGGTGGTCTTATTG | ACCTGCCCAACCATTCTATC | 102% |
| *Fmc1* (NM_025363) | TTCCAAGCTGCCACCTATC | CAACTGGAGGCCCACTAAA | 90% |
| *Rpl37* (NM_026069) | CCAAGCGCAAGAGGAAGTATAA | CATGTCTGAATCTGCGGTAGAC | 84% |
| *Sox9* (NM_011448) | GGCAAGCTCTGGAGGCTG | CCTCCACGAAGGGTCTCTTCT | 98% |
| *Xist* (NR_001463) | GCCCAAAGGGACAAACAATC | GTAGCGAGGACTTGAAGAGAAG | 134% |

Table S2: miRNA RT-qPCR primer sequences.

| **miRNA** | **Forward primer sequence (5’-3’)** | **Primer efficiency (%)** | **E-val** |
| --- | --- | --- | --- |
| *cel-miR-39* | TCACCGGGTGTAAATCAGCTTG | 57% | 1.57 |
| *mmu-miR-9-3p* | TAAGCTAGATAACCGAAAGTAAAAAA | 70% | 1.7 |
| *mmu-miR-10b-5p* | CCTGTAGAACCGAATTTGTGA | 218% | 3.18 |
| *mmu-miR-19a-3p* | TGTGCAAATCTATGCAAAACTGA | 320% | 4.2 |
| *mmu-miR-101a-3p* | TACAGTACTGTGATAACTGAAAAAAA | 68% | 1.68 |
| *mmu-miR-130b-3p* | CAGTGCAATGATGAAAGGGCAT | 180% | 2.80 |
| *mmu-miR-199a-5p* | CCCAGTGTTCAGACTACCTGTTC | 177% | 2.77 |
| *mmu-miR-200c-3p* | TAATACTGCCGGGTAATGATGGA | 266% | 3.66 |
| *mmu-miR-205-5p* | TCCTTCATTCCACCGGAGTCTGAAAA | 153% | 2.53 |
| *mmu-miR-206-3p* | TGGAATGTAAGGAAGTGTGTGG | 182% | 2.82 |
| *mmu-miR-409-5p* | AGGTTACCCGAGCAACTTTGCAT | 157% | 2.57 |
| *mmu-miR-872-5p* | AGGTTACTTGTTAGTTCAGGAAAA | 317% | 4.17 |

Table S3: Top 10 miRNAs in small RNA-seq data by combined (n = 6) read count.

| **Mature miRNA** | **Total read counts** | | **Literature search** |
| --- | --- | --- | --- |
| *mmu-miR-9-5p* | 33824856 | Highly expressed during neurogenesis to regulate various aspects of vertebrate brain development (135). | |
| *mmu-miR-125b-5p* | 18302138 | Expressed in the mouse embryonic nervous system from stage E12 (136). | |
| *mmu-miR-92a-3p* | 8007628 | Specifically expressed in glial cells in the neonatal rat brain (137). | |
| *mmu-miR-181a-5p* | 7788531 | *miR-181* family members are highly expressed in the cortex and involved with synaptogenesis. Increased expression noted in autism animal models and patients (138). | |
| *mmu-miR-99a-5p* | 5823098 | High expression in human NSC cultures (139) and associated with cell cycle regulation (140). | |
| *mmu-let-7c-5p* | 5780019 | Expression levels continuously rise from E12.0 in embryonic mouse brains and impact stem cell behavior (141). | |
| *mmu-miR-9-3p* | 5752114 | The sister strand of *mmu-miR-9-5p* has also been implicated in various facets of neural cell development (103). | |
| *mmu-miR-99b-5p* | 5144303 | See *mmu-miR-99a-5p.* | |
| *mmu-miR-26a-5p* | 4807191 | Various roles in mouse brain development including as a component of a key regulatory circuit for neural progenitor proliferation (142). | |
| *mmu-let-7a-5p* | 4735868 | See *mmu-let-7c-5p*. | |


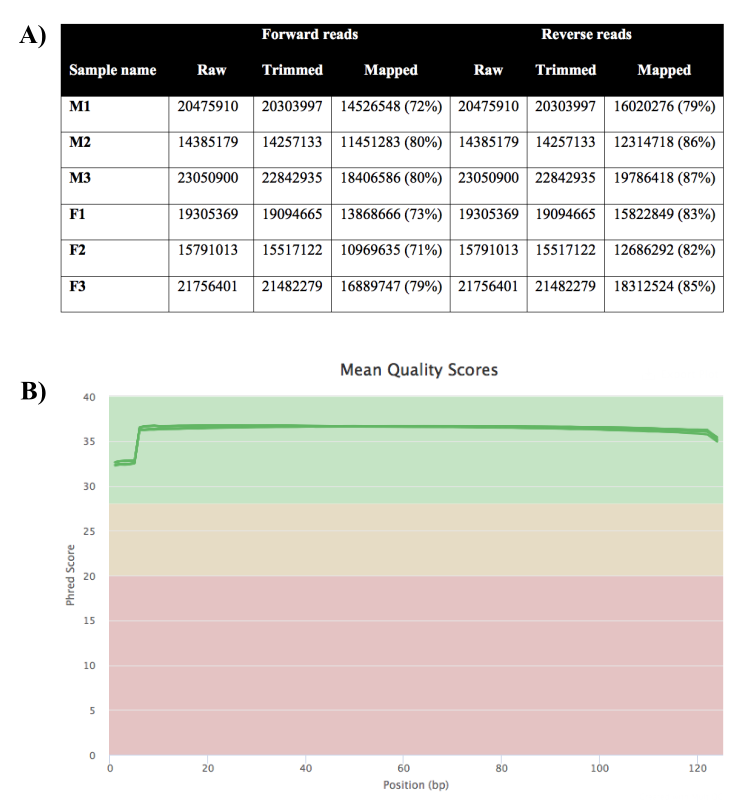


Figure S1: RNA-seq mapping statistics and read quality. **A)** Read count data for each sequenced sample in raw file, in the output from trimming and QC, and following mapping to the mm9 reference genome for both forward and reverse sequences. **B)** MultiQC plot of aggregated phred scores for all samples across the 125 bp read length.


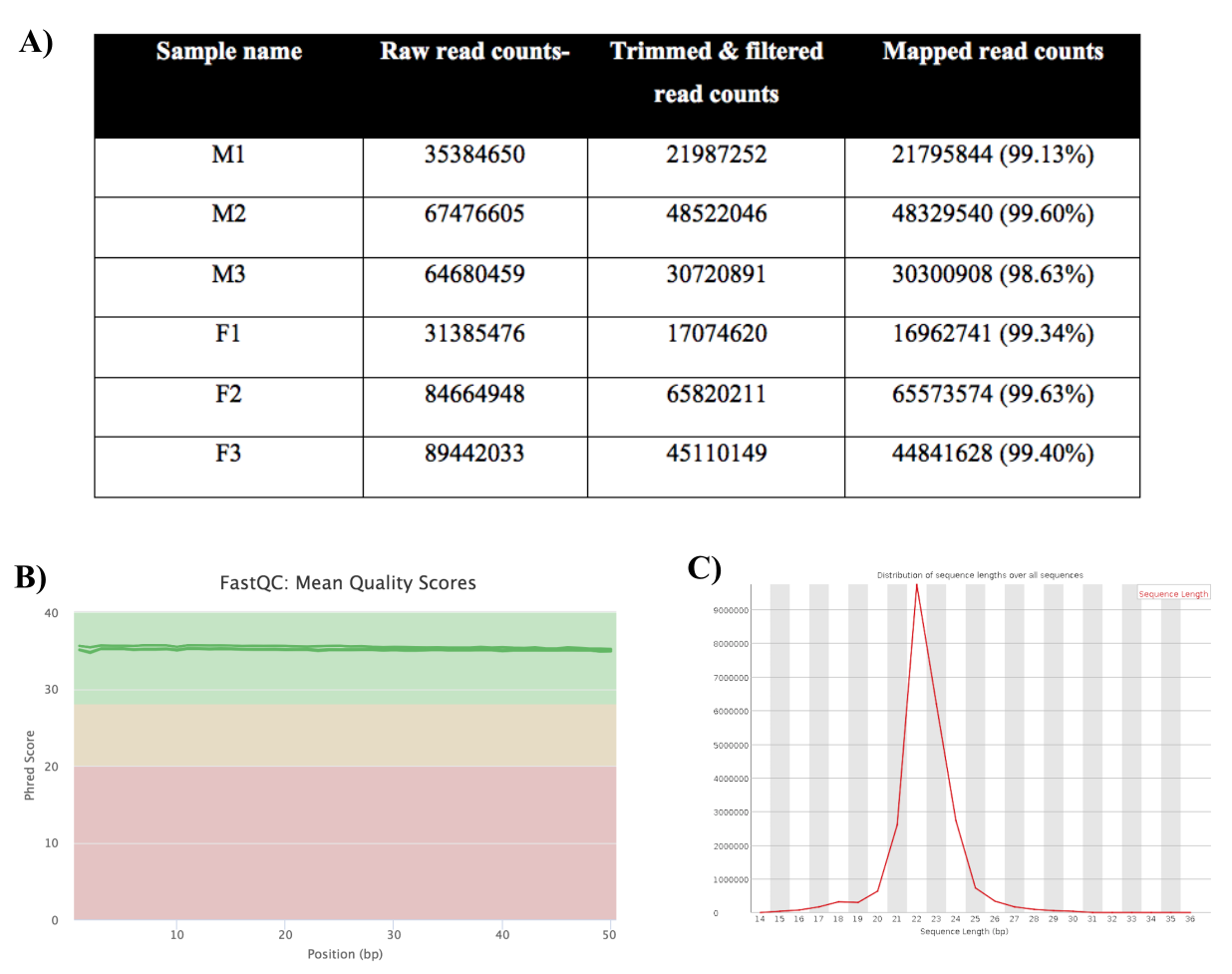


Figure S2: Small RNA-seq mapping statistics and read quality. **A)** Read count data for each sequenced sample in the raw file, in the output from trimming and QC, and following mapping to the mm9 reference genome, with the percentage of trimmed reads successfully mapped in brackets. **B)** FastQC plot of a representative sample showing phred score across the 50 bp untrimmed read. **C)** Read length distribution plot for a representative sample showing that read lengths peak at ~22 bp.

**
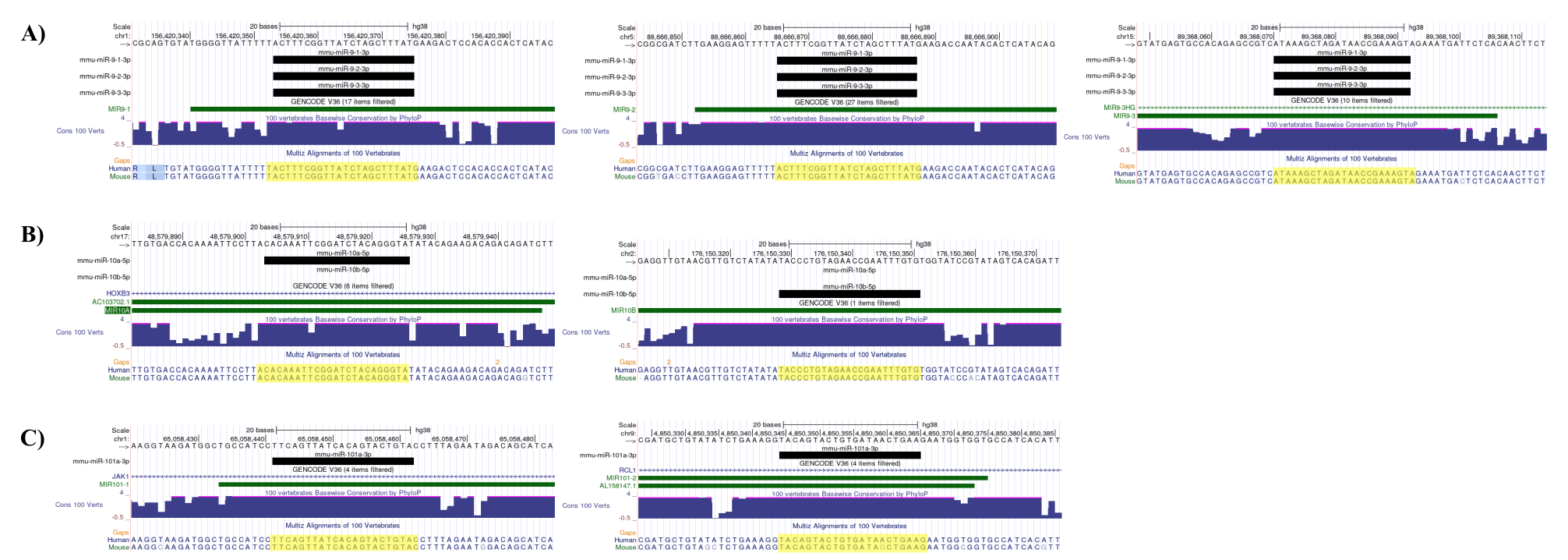
**


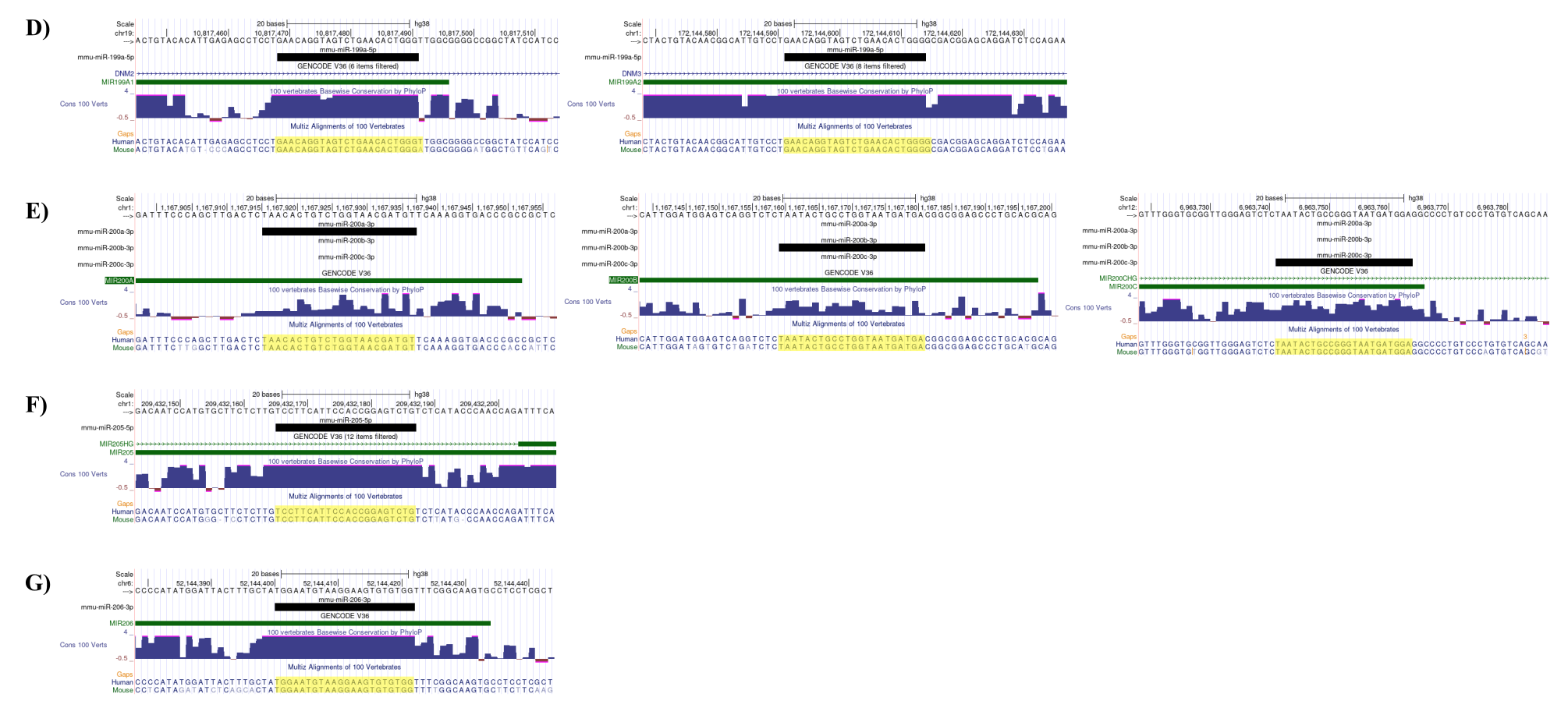


Figure S4: Conservation of sex-biased miRNAs of interest between mouse and human genomes. Genome browser screenshots depict mouse miRNAs (black) mapping to the UCSC hg38 genome (green). “Cons 100 Verts” indicates PhyloP scores across 100 vertebrates and the “Multiz alignment” track shows human and mouse sequences at base pair resolution. Sequences with 100% conservation are highlighted yellow. All paralogs have been included for the 7 miRNAs of interest: a) miR-9-3p, b) miR-10b-5p, c) miR-101-3p, d) miR-199-5p, e) miR-200-3p, f) miR-205-5p, g) miR-206-3p.


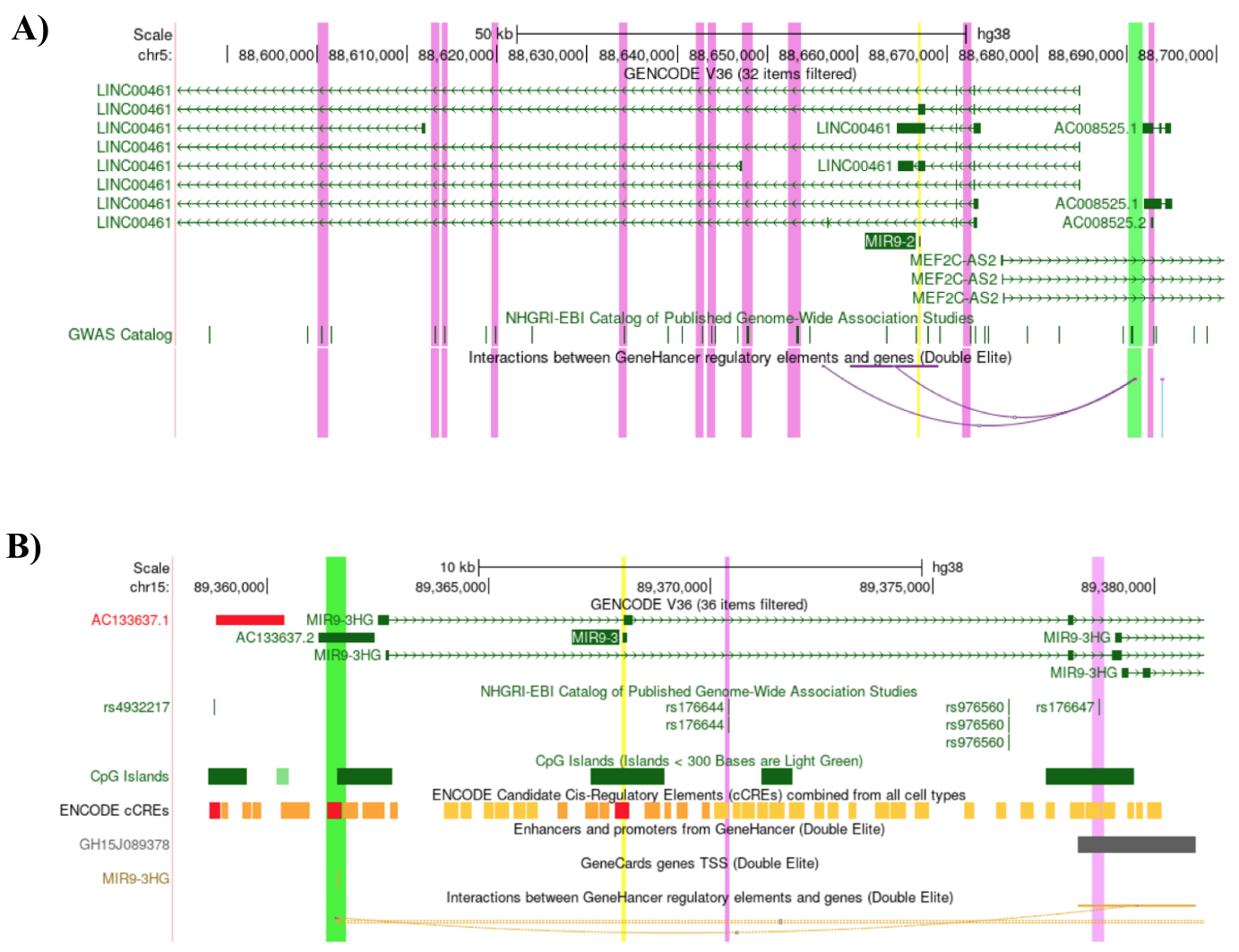


Figure S5: Genome browser screenshot from hg38 assembly showing the MIR9-2 and MIR9-3 loci, respectively. Yellow highlighting indicates the miRNA gene, pink indicates neurologically associated SNPs, and green is the TSS for each miRNA gene.
